# IRE1α Implications in Endoplasmic Reticulum Stress-Mediated Development and Pathogenesis of Autoimmune Diseases

**DOI:** 10.3389/fimmu.2018.01289

**Published:** 2018-06-06

**Authors:** Raghu Patil Junjappa, Prakash Patil, Kashi Raj Bhattarai, Hyung-Ryong Kim, Han-Jung Chae

**Affiliations:** ^1^Department of Pharmacology, School of Medicine, Institute of New Drug Development, Chonbuk National University, Jeonju, South Korea; ^2^Graduate School, Daegu Gyeongbuk Institute of Science and Technology (DGIST), Daegu, South Korea

**Keywords:** autoimmune diseases, cytokines, inflammation, IRE1α, regulated IRE1α-dependent decay, unfolded protein response signaling pathways

## Abstract

Inositol-requiring transmembrane kinase/endoribonuclease 1α (IRE1α) is the most prominent and evolutionarily conserved endoplasmic reticulum (ER) membrane protein. This transduces the signal of misfolded protein accumulation in the ER, named as ER stress, to the nucleus as “unfolded protein response (UPR).” The ER stress-mediated IRE1α signaling pathway arbitrates the yin and yang of cell life. IRE1α has been implicated in several physiological as well as pathological conditions, including immune disorders. Autoimmune diseases are caused by abnormal immune responses that develop due to genetic mutations and several environmental factors, including infections and chemicals. These factors dysregulate the cell immune reactions, such as cytokine secretion, antigen presentation, and autoantigen generation. However, the mechanisms involved, in which these factors induce the onset of autoimmune diseases, are remaining unknown. Considering that these environmental factors also induce the UPR, which is expected to have significant role in secretory cells and immune cells. The role of the major UPR molecule, IRE1α, in causing immune responses is well identified, but its role in inducing autoimmunity and the pathogenesis of autoimmune diseases has not been clearly elucidated. Hence, a better understanding of the role of IRE1α and its regulatory mechanisms in causing autoimmune diseases could help to identify and develop the appropriate therapeutic strategies. In this review, we mainly center the discussion on the molecular mechanisms of IRE1α in the pathophysiology of autoimmune diseases.

## Introduction

Autoimmune diseases are the consequences of an abnormal immune response in the form of autoantibodies and T-cells attacking the host’s body. These diseases include both tissue-specific and systemic disorders and affect approximately 3–5% of the population (1, 2). Most of the autoimmune diseases are heterogeneous in nature and are usually characterized by the expression of autoantibodies, pro-inflammatory cytokines, and autoreactive T-cells (3, 4). Many theories have suggested that a genetic predisposition is the main cause of autoimmune diseases. However, the concordance rates of autoimmune disease in monozygotic twins range from 12 to 67% (2, 5). Furthermore, in an *in vivo* study, collagen-induced arthritis in inbred mice of the single cage, which were comparable to identical twins, affected a minority (5). These studies give evidence that heredity accounts for only approximately one-third of the risk of developing an autoimmune disease, while environmental and epigenetic factors account for the majority of the risk (6). Many environmental factors, including microbial infection, chemicals exposure, free radicals, abnormal blood glucose, cholesterol, and inflammation are known to trigger autoinflammation (7–13). All of these factors are also known to induce endoplasmic reticulum (ER) stress (14, 15), indicating the possible association of ER stress to the onset of the autoimmune diseases. Further, several recent studies have shown that ER stress precedes the progression of autoimmune diseases (16–18). In addition, ER stress can lead to the upregulation of many pro-inflammatory cytokines, including TNFα, IL-1β, IFN-γ, IL-6, and IL-23 (19), which comprise the hallmark of autoimmune diseases (20). In spite of significant development has been made in the treatments using immunosuppressive or immunomodulatory agents, the prognosis is still poor for many patients in terms of a long-term cure (5). Therefore, clear knowledge on the mechanisms that are responsible for dysregulation of the immune system, which in turn leads to autoimmune disease, will help in developing therapeutics. Additionally, knowledge about the precise causes for the elicitation of the autoimmune response, especially ER stress-mediated immune response is required for developing treatment modalities, but these causes are still unclear.

## How Does ER Function Contribute to Autoimmune Diseases?

The ER controls multiple cellular functions involving protein folding, post-translational modifications (PTMs), fatty acid biosynthesis, detoxification, and also stores the intracellular calcium (21). About one-third of cellular proteins majorly including secretory and transmembrane proteins reach maturation in the ER (22, 23). Once ribosomes translate the mRNA, the synthesized peptide is inserted into the ER based on its signal sequence. The signal sequence is then cleaved, and the protein is moved into the lumen of the ER. Inside the lumen, it is folded into its functional conformation and remains in the ER or, through the Golgi bodies, is transported to other cellular organelles or cytoplasmic membrane or is secreted. However, regardless of its destination, newly synthesized proteins undergo various processes in the lumen of the ER (24). These processes include folding, formation of multisubunit complexes, disulfide bond formation, N-glycosylation, and many other PTMs (25). In addition, the ER has been implicated in metabolism of glucose, lipids, and cholesterol, and also in the process of autophagy (22).

As the functions of the ER required, the environment in the ER is oxidative and rich in calcium and other protein folding machineries (26). The protein folding requirement and degree of secretory protein synthesis vary across cell types. Cells with secretory functions, such as pancreatic β cells and liver cells are rich in ER to meet the high, fluctuating demand (27, 28). Inside the ER, secretory proteins are folded precisely to their native conformations with the assistance of chaperones and protein disulfide isomerases (PDI), and then the properly folded proteins translocate to their destined place based on the signal sequence (21, 29). However, cells can encounter conditions, such as viral infections, cancers, neurodegenerative diseases, diabetes, inflammation, a high demand of secretory proteins, and other aberrations at the cellular level during which ER protein folding functions can be disturbed (30). This can result in the accumulation of unfolded proteins inside the ER, entitled as ER stress (30–32). However, cells have developed a mechanism to sense these changes and try to reestablish homeostasis by stimulating specific signal transducing pathways, named as the unfolded protein response (UPR) (33, 34). This process is well conserved from yeast to humans (35).

The UPR system initially tries to restore homeostasis through transcriptional induction of folding enzymes, chaperones, oxidoreductases, reduced translation, autophagy, lipid biogenesis, vesicular trafficking, degradation of ER-associated mRNA, and degradation of unfolded proteins through ER-associated protein degradation (ERAD) (36). However, this adaptive process may fail, due to persistent stress resulting from a high demand for proteins, especially in secretory cells, and due to chronic diseases (37). In that case, the activated UPR transforms it signals from survival to a death inducing pathway to clear the affected cells from the system (38). However, unrestricted apoptosis leads to a loss of cells in organs (38, 39). These pro-death signaling pathways cause the pathogenesis of many diseases through increasing reactive oxygen species (ROS), activating proapoptotic proteins, and activating inflammatory molecules (40).

In addition to the secretion of inflammatory cytokines, disturbances in the ER environment result in abnormal PTM of many proteins, which can activate the autoimmune response by developing into neoantigens (41). The ER stress-mediated generation of autoantigens/neoantigens is reviewed elsewhere (42, 43). Indeed, several ER proteins, including insulin, glucose-regulated protein 78 (GRP78), glutamic acid decarboxylase 65, and chromogranin A are turned into neoantigens due to abnormal PTM (41, 44, 45). These neoantigens activate autoreactive T-cells, which leads to pathological conditions (46). Furthermore, in rat insulinoma (INS-1E) cells and non-obese diabetic (NOD) mice, cytokine-induced ER stress produces the post-translationally modified chaperone protein GRP78 or immunoglobulin binding protein (BiP) (47, 48). This modified GRP78 generates autoreactive T-cells with higher levels of IL-17, TNFα, and IFN-γ production (17, 49). The cytokine-mediated calcium depletion in the ER also activates the cytosolic calcium-dependent PTM enzymes transglutaminase 2 (Tgase2) and peptidylarginine deiminases, which generate the neoantigens (50). In addition, ER stress-mediated UPR upregulates the production of the important pro-inflammatory cytokines, such as IL-1β, TNFα, IL-17, and IL-23, which further enhance the tissue damage (51). These cytokines are known to contribute significantly in the pathogenesis of autoimmune disorders (52, 53). Interestingly, cytokines, in a feedback loop mechanism can induce ER stress and apoptosis through the UPR (54). Taken together these discoveries imply the contribution of ER stress to the development of autoimmune diseases. In this review, we center on the implications of the conserved ER stress-transducing molecule IRE1α in the onset and pathogenesis of autoimmune diseases. We especially consider its role in immune cells and its signaling pathways in the immune response, along with potential IRE1α targeting therapies to treat autoimmune diseases.

## IRE1α/ERN1 (Inositol-Requiring Enzyme1/Endoplasmic Reticulum to Nucleus1)

IRE1α, the most evolutionarily conserved ER membrane protein regulates, many cellular processes involving cell survival and cell death (55–57). The IRE1 gene was first identified in yeast in the search of genes involved in the metabolism of inositol phospholipids; it complemented a yeast mutant requiring exogenous inositol for its growth (58). Later, from the benchmark work of Peter Walter and Kazutoshi Mori, IRE1 was identified as a UPR molecule through the screening of yeast genes involved in signal transduction from the ER to the nucleus during misfolded protein accumulation (59, 60). In metazoans, IRE1 exists in two isoforms: IRE1α/ERN1 and IRE1β/ERN2. IRE1α is localized to the ER membrane and has an N-terminal signal-sensing ER luminal domain (LD), a type I transmembrane domain (TD) and a dual enzymatic, hydrophilic, cytosolic C-terminal domain having both kinase and endoribonuclease functions (61). IRE1α is prevalent in almost all tissues, but IRE1β is expressed only in intestinal epithelial cells (IECs) (62) and airway mucous cells (63). The amino acid sequence of the sensor, kinase, and RNase domains (RDs) of human IRE1α and IRE1β have 48, 80, and 61% identity, respectively (64).

### Activation Mechanism of IRE1α During ER Stress and Its Downstream Signals

**Figure 1:**
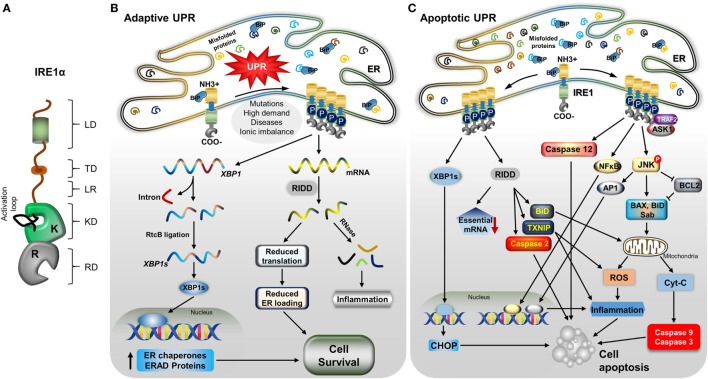
IRE1α structure and its downstream mechanisms in mild and severe stress. **(A)** A model depicting the IRE1α structure and its functional domains: luminal domain (LD), transmembrane domain (TD), linker region (LR), kinase domain (KD), RNase domain (RD), and an activation loop at the KD. **(B)** Stress factors, such as mutations, high demand for secretory proteins, ionic imbalance, and disease cause an accumulation of misfolded proteins, leading to activation of the unfolded protein response. Immunoglobulin binding protein regulates IRE1α through dimerization, autophosphorylation, and further oligomerization. During mild endoplasmic reticulum (ER) stress, activated IRE1α helps the cells to recover from stress through increasing the ER folding chaperones and ER-associated degradation (ERAD) components by generating stable transcription factor XBP1s, as follows: active IRE1α cleaves *XBP1* mRNA, and the cleaved fragments are ligated by RtcB. Stable XBP1 mRNA is generated and translated to form the transcription factor XBP1s, which moves to the nucleus and induces the expression of chaperone proteins and ERAD process-associated genes. Another process, regulated IRE1α-dependent decay (RIDD), degrades mRNAs and reduces the load of new proteins entering ER, which helps in cell survival; in addition, the generation of small RNA fragments triggers the inflammatory response. **(C)** With severe stress, IRE1α induces the alternative pathway: apoptotic signaling by recruiting TNF receptor-associated factor 2 (TRAF2) and apoptosis signaling kinase1, leading to c-Jun N-terminal kinase (JNK) phosphorylation. Phosphorylated JNK induces apoptosis through many signaling pathways. It causes the proapoptotic proteins Bcl-2-associated X protein (BaX), and Bim to translocate to the mitochondrial membrane by inhibiting antiapoptotic B-cell lymphoma family 2 (Bcl-2). The mitochondrial membrane is ruptured, and cytochrome C is released, which activates caspase-9 and -3, which in turn cleave many proteins and cause cell death. Phosphorylated JNK also translocates Sab protein, which increases the mitochondrial reactive oxygen species (ROS) and leads to cell death directly, as well as ROS inducing inflammation-mediated cell death. In addition, JNK-mediated transcription factor AP-1 induces pro-inflammatory cytokine-mediated cell death. IRE1α also can induce inflammation-mediated cell death by activating NFκB. Furthermore, RIDD activity, which degrades prosurvival mRNAs, which increases caspase-2 expression, causes translocation of BH3-interacting domain to the mitochondria, and also degrades miRNA17, which stabilizes the thioredoxin-interacting protein thioredoxin-interacting protein, leading to inflammation and ROS-mediated cell death. In addition, spliced XBP1s stimulates the expression of the proapoptotic protein CCAAT-enhancer-binding protein homologous protein (CHOP), which induces cell death. Finally, IRE1α/TRAF2 association can activate caspase-12-mediated cell apoptosis.

The disturbed environment in the ER during pathological conditions and also at a low level in regular physiological conditions, leads to the activation of IRE1α (65) (Figure 1A). In normal conditions, it is negatively regulated by the attachment of the ER chaperonic protein BiP, on the ER LD of IRE1α (66, 67). However, during the accumulation of misfolded proteins, BiP separates and binds to the misfolded proteins due to its higher affinity for these proteins than IRE1α (68). The dissociation of BiP leads to self-association of IRE1α’s LD, causing IRE1α to dimerize and trans-autophosphorylate its cytoplasmic kinase domain (69). This leads to conformational change in the RD, which then becomes enzymatically active, and also forms higher order oligomers (69–73). It was reported recently that misfolded proteins can also bind directly to IRE1α and similarly activate it (74). In addition, membrane aberrancy, alteration in the cellular lipid composition and membrane lipid saturation also activate IRE1α through its TDs (75–77). Upon the activation of its kinase and endoribonuclease functions, IRE1α takes out an intron of 26 nucleotide length from X-box binding 1 (*XBP1*) mRNA by an unconventional method in the cytoplasm: specifically, in a spliceosome-independent manner, leading to introduction of a new termination codon due to a frame-shift in the coding sequence (78, 79). The IRE1α endoribonuclease activity gives rise to two free ends of 2′3′-cyclic phosphate and 5′-OH at 5′ and 3′exons, respectively (80). However, this endoribonuclease activity depends on the presence of a specific pair of appropriate stem-loop structures and a conserved consensus sequence, CNCNNGN (where N is any base) in the mRNA (55, 81). These ends are ligated by tRNA ligase, RtcB, generating the stable transcription factor XBP1 (XBP1s) (82) (Figure 1B). XBP1s targets many genes involved in multiple cellular functions and this activity varies with cell type and condition (83). In particular, XBP1s induce the expression of proteins involved in ER stress attenuation: protein folding chaperone GRP78, PDI, and translocation proteins (84, 85). In addition, XBP1s promotes the expression of the ER quality-control proteins heat shock protein 40 kDa (DnaJ), p58, ER-resident molecule (ERdj4), ER degradation-enhancing α-mannosidase-like protein (EDEM) involved in ERAD, and ER-to-Golgi transport components (81, 86–88). Further, XBP1s is necessary for basic physiological functions, mainly in secretory and differentiating cells (89–91), and also contributes to inflammation (92). IRE1α is also inevitable for the placental and embryonic development. Lacking of IRE1α in mice resulted in embryonic mortality during gestation, due to liver hypoplasia and reduced angiogenesis (93).

In addition to the generation of a stable XBP1s transcription factor, IRE1α activation causes the cleavage of other ER-localized mRNAs, cytosolic mRNAs, ribosomal RNA, and miRNAs, leading to their degradation in a process named as regulated Ire1-dependent decay (RIDD) (94–97) (Figure 1B). This cleavage activity is also sequence specific, but it does not necessarily require a double-loop structure, unlike in XBP1 splicing (55, 56). RIDD function helps in ER stress reduction, due to decreasing in the inflow of newly synthesized proteins into the ER, and it also participates in biological functions, such as glucose metabolism, inflammation, and apoptosis (55). In addition, cleaved mRNA fragments generate an inflammatory response (98). Notably, RIDD is hyperactivated under conditions of XBP1 deficiency and is implicated in both cell survival and death depending on the nature of tissue and level of stress intensity (19, 99, 100). Interestingly, another isoform, IRE1β, is primarily involved in RIDD activity but is less active in generating XBP1s compared with IRE1α and mediates the protein-folding process in lung and gut epithelial secretory cells (63, 101).

### IRE1α-Mediated Cell Apoptosis Pathways Involved in Pathogenesis

IRE1α induces the cell death pathway by activating different apoptosis-inducing molecules through its endonuclease and kinase functions (Figure 1C). However, this action of IRE1α is highly controlled or restricted, depending on the level and type of stress or tissue origin (102, 103). IRE1α activity is necessary for the normal functions of the cells and also for the stress adaptive process (104). However, when a threshold reached in terms of the balance of survival and death signals, IRE1α activates the cell death signaling, which is regulated by its regulating partner molecules (34, 105). IRE1α triggers cell death by promoting the intrinsic apoptosis pathway through interaction with a hub of diverse molecules through TNF receptor-associated factor 2 (TRAF2) (40). IRE1α and TRAF2 association forms a complex with apoptosis signaling kinase 1 (ASK1) and phosphorylate c-Jun N-terminal kinase (JNK) (106–108). The phosphorylated JNK induces the apoptotic signal through many pathways. Sustained JNK activation is known to trigger apoptosis by regulating specific proteins of the B-cell lymphoma family 2 (Bcl-2) (40, 109, 110), which activates the cytochrome C-mediated apoptotic pathway. JNK translocates to the mitochondrial membrane and promotes apoptosis by phosphorylating and inhibiting the anti-apoptotic Bcl-2 proteins (111–113). In addition, it promotes the localization of the Bcl-2-associated X protein (BaX) and Bcl-2-associated death promoter (BaD) proapoptotic proteins to the mitochondria; this damages the mitochondrial membrane, leading to release of cytochrome C, which consequently activates the caspase-9 and -3, inducing cell death. Additionally, BaD can antagonize anti-apoptotic Bcl-2 proteins, thus promoting apoptosis (110, 111, 113). Furthermore, activated JNK binds to Sab (SH3 homology-associated BTK binding protein) on the outer mitochondrial membrane, which promotes mitochondrial ROS production and induces cell death (114). JNK activation also mediates cell death through AP-1-mediated expression of pro-apoptotic genes (115–117). IRE1α/TRAF2 association is also implicated in the induction of the pro-apoptotic signaling pathway through caspase-12 activation (118, 119). Interestingly, receptor-interacting serine/threonine protein kinase 1 (RIPK1) stimulates the IRE1α-mediated JNK activation *via* a TNF-independent interaction of tumor necrosis factor receptor 1 on the ER membrane (120, 121). The association of receptor-interacting serine/threonine protein kinase 1 (RIPK1) and IRE1α also promotes death receptor-independent caspase-8 activation, which then induce cell death through activating caspase-9 and caspase-3-mediated cell damage. Additionally, the IRE1α/TRAF2 interaction promotes NFκB-dependent autocrine production of TNFα and apoptosis (122, 123). In addition, IRE1α/XBP1s also induce apoptosis of hepatocytes in an ER stress-dependent manner by inhibiting Akt through increasing Pleckstrin homology like domain family A member 3 (PHLDA3) expression (124). In addition, XBP1s also enhance CCAAT-enhancer-binding protein homologous protein (CHOP)-mediated cell death (125).

Furthermore, the IRE1α-mediated RIDD process has also been implicated in cell apoptosis (55). During hyper-activation, IRE1α degrades cell-essential mRNA, which leads to reduced survival. RIDD activity also contributes to mitochondrial apoptotic pathway through caspase-2 and BH3-interacting domain activation by degrading the caspase-2 repressing miRNA, resulting in enhanced expression and activation of caspase-2 (126, 127). In addition, IRE1α degrades miRNA-17, a repressor of thioredoxin-interacting protein (TXNIP), resulting in TXNIP-mediated activation of the nucleotide-binding domain, leucine-rich-containing family, pyrin domain-containing-3 (NLRP3) inflammasome, and its caspase-1 and caspase-2-dependent pro-death pathways (128, 129).

## IRE1α in Immune Cells

The role of IRE1α in immune functions is being progressively identified as a possible mechanism for multiple complex immune-related diseases (130). Primarily, IRE1α plays a very important role in the survival and functioning of many immune cell types (131). Due to their secretory function, immune cells have larger ER with higher protein-folding activity, and consequently they are more susceptible to agents, such as toxins, diseases, and pathogens that induce ER stress (132). This may necessitate the presence of IRE1α in immune cells. In addition to the pathophysiological effects, IRE1α activation plays an important function in the normal physiology of immunologically important cell types. The functional requirements of IRE1α in different immune cell types are summarized in Table 1.

**Table 1:** IRE1α functions in different immune cells.

Immune cell	Functions of IRE1α	Reference
B cells and plasma cells	IRE1α and XBP1s are required for the terminal differentiation of B cells to plasma cells IRE1α is required for the expansion of the endoplasmic reticulum (ER) and antibody production and secretion during both physiological and pathological immune responses	(53, 89, 133, 134)
T cell	IRE1α and XBP1s are active in early stages of T-cell, in bone marrow pro-B cells, CD4^+^ T-cells, CD8^+^ thymic T-cells, CD8^+^ splenic T-cells, and cytotoxic T-cell XBP1s is also required for the terminal differentiation of CD8^+^-effector T-cells Active XBP1s was found in CD8^+^ T-cells in acute infection with *Listeria monocytogenes*	(135–139)
Dendritic cells (DCs)	IRE1α and XBP1s were found constitutively active in DCs Loss of XBP1s leads to significantly reduced numbers of both conventional and plasmacytoid DCs XBP1s deficiency in DCs increased the rate of apoptosis IRE1α/XBP1s-induced lipid accumulation in DCs impaired MHC class I-mediated antigen presentation to cytotoxic T-cells Loss of XBP1s in splenic type 1 conventional dendritic cells (cDC1s) resulted in functional alterations, but also impaired the survival of mucosal cDC1s Loss of XBP1s in CD8α^+^ DCs led to defects in phenotype and MHC class I antigen presentation	(135, 139–142)
Granulocytes (eosinophils)	Hematopoietic deletion of XBP1s in mice led to the loss of fully mature eosinophils Loss of XBP1s specifically in eosinophils led to a significantly smaller pool of eosinophils in the bone marrow and reduced eosinophil differentiation XBP1s is needed for the sustained viability of eosinophils	(143)
Macrophages	IRE1α and XBP1s are crucial for optimal and sustained production of pro-inflammatory cytokines in macrophages Macrophage-specific loss of XBP1 impairs the production of IL-6, TNFα, IFN-β, IL1-β, and C-C motif chemokine ligand 2 (CCL2) IRE1α functions in macrophage polarization	(144, 145)
Hematopoietic cells	IRE1α and XBP1s play a role in the cell cycle and differentiation of hematopoietic cell	(146)
Monocytes	XBP1s and its downstream chaperone immunoglobulin binding protein are involved in the differentiation of monocytes into macrophages The IRE1α/XBP1s pathway has importance in the development of monocytes into osteoclasts in response to RANKL	(147, 148)
Neutrophils	IRE1α function is required for neutrophil infiltration Knockdown of XBP1 in neutrophils impaired the release of granules	(149)
Natural killer (NK) cell	Expression of XBP1s was observed in the initiation of NK cell-mediated direct cytotoxicity or antibody-dependent cell-mediated cytotoxicity (ADCC) in leukemia or lymphoma target cells Pharmacological inhibition of the IRE1α/XBP1s pathway significantly impaired both NK cell-mediated direct-cytotoxicity and antibody-dependent cell-mediated cytotoxicity (ADCC). This indicates that XBP1s is essential for optimal NK cell cytotoxicity	(150)
Mast cells	IRE1α may involve mast cell functions. Since, application of IRE1α-specific inhibitor, 8-formyl-7-hydroxy-4-methylcoumarin, in mast cells reduced the IgE-mediated degranulation of mast cells as well as release of cytokines, such as TNF-α and IL-4	(151)
Paneth cells	XBP1s is necessary for Paneth cell development in the gut XBP1 deletion caused Paneth cell dysfunction and increased susceptibility to enteritis and induced colitis in intestinal epithelial cells	(92)

### IRE1α-Mediated Immune Response

**Figure 2:**
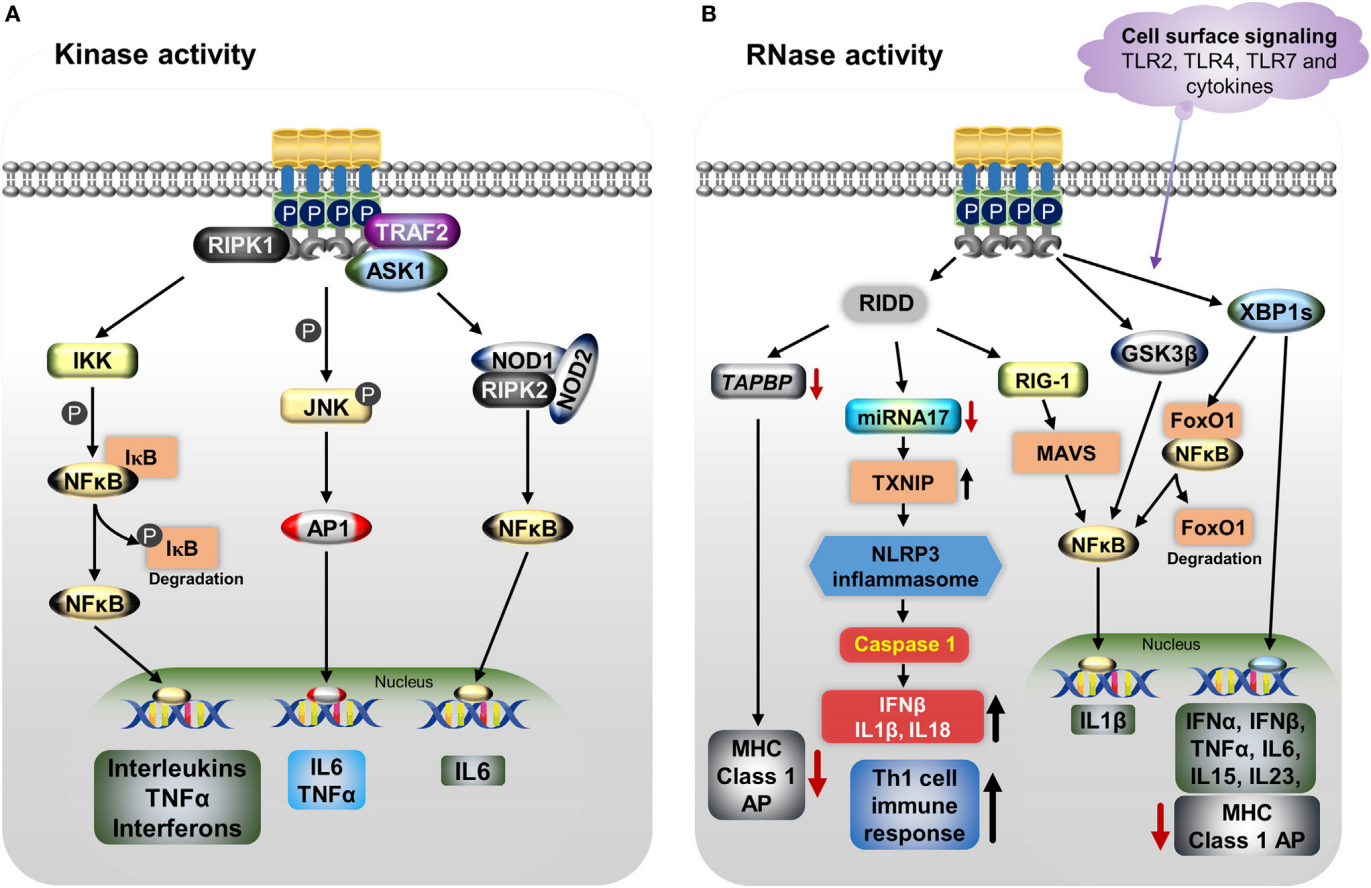
IRE1α regulates the immune function through various mechanisms including both its kinase **(A)** and RNase **(B)** functions. **(A)** Endoplasmic reticulum (ER) stress-activated, serine/threonine-protein kinase/endoribonuclease IRE1α binds to TNF receptor-associated factor 2 (TRAF2), apoptosis signaling kinase1 (ASK1), and receptor-interacting serine/threonine protein kinase 1 (RIPK1), resulting in phosphorylation of c-Jun N-terminal kinase. Then c-Jun interacts with c-Fos forms the active transcription factor AP-1, and increases the production of IL-6 and TNFα. Furthermore, the IRE1α/TRAF2/ASK1 complex activates the inhibitory kappa B kinase (IKK), which phosphorylates inhibitor of kappa B (IκB), leading to release of NFκB and its translocation to the nucleus, where it induces the expression of cytokines. The dissociated IκB is then degraded by proteasomes. The IRE1α–TRAF2 complex increases IL-6 production *via* the association of nucleotide-binding oligomerization domain (NOD)-containing proteins 1 and 2 (NOD1 and NOD2) and receptor-interacting serine/threonine-protein kinase 2 (RIPK2). **(B)** IRE1α through its RNase function generates splices—X-box-binding protein 1 (XBP1s) transcription factor induces the expression of several pro-inflammatory cytokines and also decreases MHC class I antigen presentation. In addition, XBP1s increases NFκB nuclear translocation by mediating the degradation of FoxO1, an inhibitor of NFκB. Furthermore, IRE1α activation differentially regulates the expression of the pro-inflammatory cytokine IL-1β gene *via* activation of glycogen synthase kinase-3β. The regulated IRE1α-dependent decay (RIDD) degrades miR-17, leading to an increase in thioredoxin-interacting protein expression. This in turn activates nucleotide-binding domain, leucine-rich-containing family, pyrin domain-containing-3 inflammasome activity, which leads to procaspase-1 cleavage, which subsequently activates IL-1β, IL-18, and IFN-β, and also increases the Th-cell 1 immune response. RIDD generates small fragments of RNA, which activate the retinoic inducible gene-I and mitochondrial antiviral protein, increasing IFNβ production *via* NFκB. In addition, RIDD reduces the *TAPBP* mRNA level, leading to decreased antigen presentation. Toll-like receptors 2, 4, and 7 and other cytokines can directly activate the IRE1α/XBP1s pathway without ER stress and cause the release of many pro-inflammatory cytokines.

Endoplasmic reticulum stress, as well as cytokine-mediated IRE1α activation with its kinase and RNase properties, triggers the immune response through various downstream pathways depending on the tissue types. These pathways are involved in pathogenesis of various diseases (Figure 2). Activation of IRE1α in immune cells and other stromal cells induces the secretion of many cytokines, such as IL-1β, IL-6, IL-23, IFN-β, and TNFα (40, 172, 173). The kinase function of IRE1α in association with TRAF2 mediates phosphorylation of JNK (p-JNK). Then, p-JNK interacts with Fos and forms the AP-1 transcription factor (115), which increases the gene expression of pro-inflammatory cytokine IL-6 (40, 174). In addition, IRE1α can activate the pattern recognition receptors, which include the nucleotide-binding oligomerization domain containing proteins 1 and 2 (NOD1/2). This causes the release of IL-6 through receptor-interacting serine/threonine-protein kinase 2 (RIPK2) (175). Furthermore, activated IRE1α triggers IκBα kinase (IKK)-mediated phosphorylation of IκBα (the inhibitor of NFκB), leading to its degradation, NFκB activation, and further release of TNFα and interleukins (ILs) (122, 176, 177).

The RNase activity of IRE1α causes the release of pro-inflammatory cytokines through both the XBP1s and RIDD pathways. The IRE1α/XBP1s pathway is activated during TLR3, TLR4, and TLR7 ligand stimulation during pathogenesis inducing the type I interferons (IFNs), IFN-α and IFN-β genes expression and furthering the pathogenesis of autoimmune and inflammatory diseases (178, 179). In one study, knockdown or inhibition of IRE1α as well as XBP1 reduced the production of IL-1β in primary airway epithelial cells and the production of IL-1β along with the chemokine, C-C motif chemokine ligand 2 (CCL2), in macrophages (145, 180). In a study of apolipoprotein E (ApoE) knockout mice, IRE1α inhibition with 8-formyl-7-hydroxy-4-methylcoumarin (4μ8c) markedly suppressed the T-helper-1 (Th-1) immune responses, as evidenced by decreased IFN-γ (145). This outcome was mediated through the inhibition of the NLRP3 inflammasome, which otherwise stimulates the secretion of IL-1β and IL-18, cytokines known to generate Th-1-type immune responses (145, 181–183); this might have implications toward the autoimmune response (181). In addition, in a study of dendritic cells (DCs), loss of XBP1 led to the reduction of IFN-α production in response to treatment with the TLR2 agonist CpG, causing the ER stress-associated cell death (135). Furthermore, XBP1s also stimulate the expression of the pro-inflammatory cytokines, such as IL-6, IL-15, and TNFα in splenic cells, multiple myeloma cells, and macrophages (51, 134, 144, 167, 184). Interestingly, IRE1α activation differentially regulates the expression of the pro-inflammatory cytokine IL-1β gene *via* activation of glycogen synthase kinase-3β (51).

In addition to XBP1s, IRE1α’s RIDD activity triggers the production of type I IFNs. The RIDD generates single-strand mRNA fragments that lack 5′caps or 3′ poly (A) tails; these fragments activate retinoic inducible gene-I (RIG-I) protein. Further, RIG-I activates the cell-autologous inflammatory response through the mitochondria-associated antiviral system producing, IFN-β and other cytokines *via* the IFN and NFκB pathways, respectively (98, 185). Further, RIDD action causes sterile inflammation and apoptosis by increasing TXNIP mRNA stability *via* degradation of the TXNIP destabilizing microRNA miR-17 (128). This leads to an increase in the TXNIP protein level, which is known to activate the NLRP3 inflammasome, leading to caspase-1 activation through procaspase-1 cleavage and then production of IL-1β and IL-18 (128, 186). The NLRP3 inflammasome-mediated immune response has been identified in various autoinflammatory and metabolic diseases (187). However, in some cases, many circulating pro-inflammatory cytokines, such as IL-1, IL-6, IL-8, and TNFα, trigger ER stress-mediated IRE1α activation (188).

Furthermore, both XBP1s and the RIDD activity of IRE1α play roles in conventional dendritic (cDC) cells and MHC class-I antigen presentation. IRE1α-induced XBP1s in airway epithelial cells increase miRNA-346, which inhibits the translation of antigen peptide transporter 1 (TAP1), a necessary component for the MHC class I subunits and peptides assembly (189, 190). The reduction of TAP1 affects the MHC class I linked antigen presentation during ER stress or disease pathogenesis (191, 192). In addition, RIDD activity inhibits the MHC class I antigen presentation in CD8^+^ cDCs by degrading the crucial component of the MHC class I machinery: transporter associated with antigen processing binding protein (TAPBP) mRNA (139). These functions of IRE1α indirectly affect the activity of CD8^+^ T-cells, which recognize MHC class I peptides during the cytotoxic process.

### Implications of IRE1α in ER Stress-Mediated Autoimmunity

**Figure 3:**
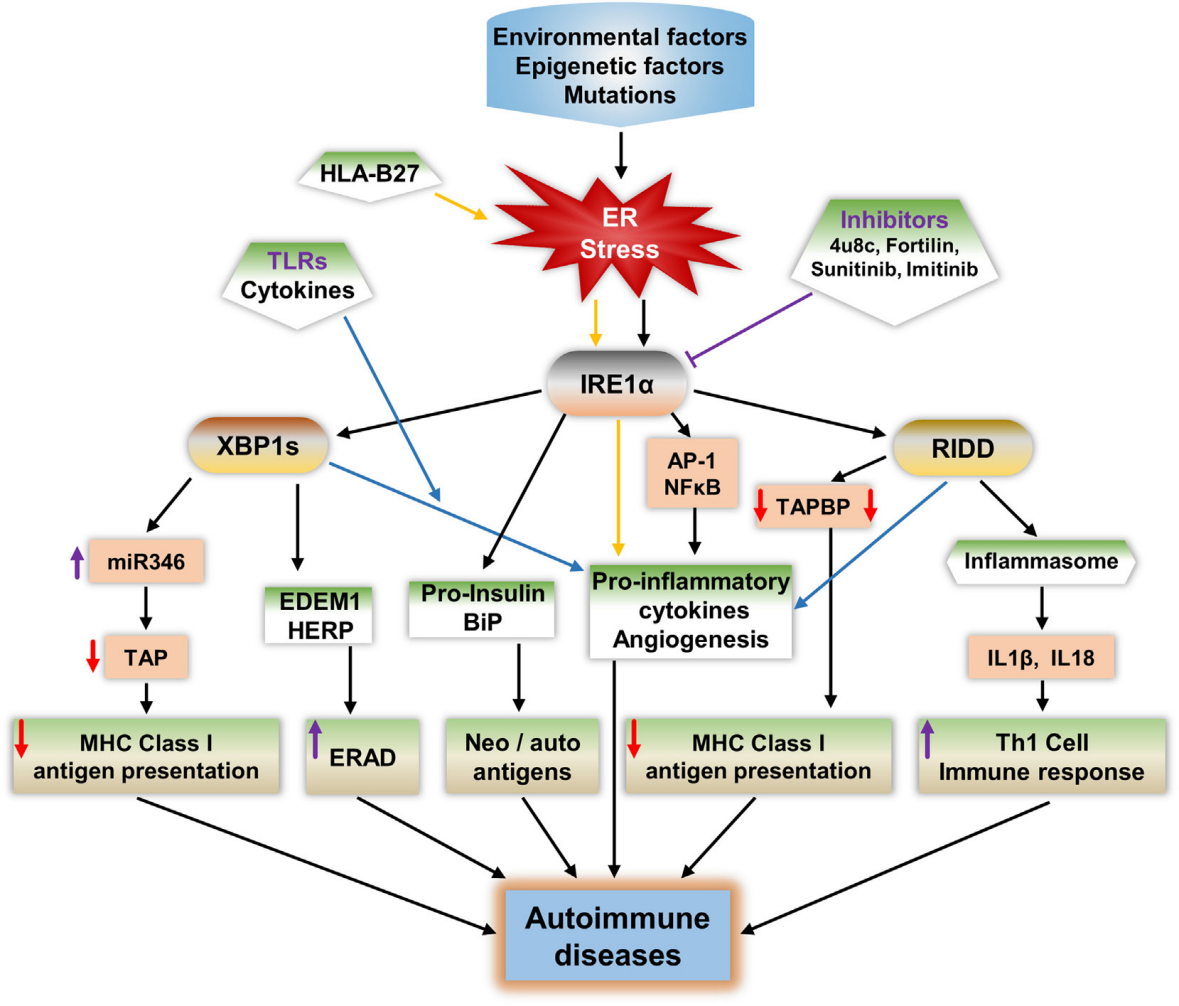
Potential mechanisms of IRE1α in the development of autoimmune diseases. IRE1α activation by environmental factors or gene mutations that induce endoplasmic reticulum (ER) stress can lead to autoimmune disease development through various pathways. Spliced XBP1s increases the expression of the microRNA miR-346, which binds to the 3′-UTR of transporter associated with antigen processing (TAP) mRNA, leading to TAP mRNA decay. This reduces MHC class I complex formation and antigen presentation. XBP1s increases the expression of ER degradation-enhancing α-mannosidase-like protein and homocysteine-induced ER protein, leading to enhanced ER-associated degradation (ERAD), which can lead to autoimmune disease by increasing immune cell survival especially that of fibroblast-like synoviocytes. Misfolded proteins may act as autoantigens; for example, human leukocyte antigen B27 (HLA-B27), immunoglobulin binding protein (BiP), and pro-insulin. IRE1α has a role in the increased expression of BiP, and pro-insulin during stress and these proteins may act as autoantigens/neoantigens. IRE1α activation during the response to misfolded HLA-B27 misfolded response may contribute to autoimmunity in ankylosing spondylitis. ER stress or toll-like receptor-activated IRE1α mediates the production of pro-inflammatory cytokines through c-Jun N-terminal kinase, such as NFκB, XBP1s, and regulated IRE1α-dependent decay (RIDD), which increases the pathogenesis in autoimmune diseases. RIDD activity reduces MHC class I antigen presentation by reducing TAPBP protein synthesis. In addition, RIDD-mediated activation of nucleotide-binding domain, leucine-rich-containing family, and pyrin domain-containing-3 inflammasomes leads to increased secretion of IL-1β and IL-18, which increase the T-helper-1 cell immune response, which is characteristic of many autoimmune diseases. Furthermore, through inhibition of IRE1α, either with small chemical molecules, such as 8-formyl-7-hydroxy-4-methylcoumarin (4μ8c), sunitinib, imatinib, or by enhancing expression of negative regulators of IRE1α such as fortilin, it may be possible to reduce the progression of autoimmune diseases.

IRE1α plays a major role in ER stress-mediated autoimmunity development possibly through five different mechanisms (Figure 3): including misfolded proteins identification by autoreactive immune cells, peptides released from apoptotic cells acting as neoantigens/autoantigens, disturbed immune-tolerance mechanisms increases ERAD-associated proteins that give the survival advantage to autoreactive cells (193), and reduced antigen presentation.

The first mechanism, in which misfolded proteins can generate an immunogenic response, has been observed in Akita mice, an experimental model for the autoimmune disease type 1 diabetes (T1D). A point mutation (Ins2, C96Y) caused pro-insulin to be misfolded and to accumulate in the pancreatic β cells, which led to UPR activation, inflammation, and ultimately β cell apoptosis (194, 195). Notably, IRE1α has a crucial function in the generation of the Akita mouse phenotype: inhibition of IRE1α’s kinase function mitigates the disease phenotype (196). Furthermore, in ankylosing spondylitis, the human leukocyte antigen B27 (HLA-B27) protein is prone to be misfolded, even under physiological conditions (197, 198). The abnormally folded HLA-B27 is involved in autoimmune diseases in two ways: either it is expressed on the cell surface as an autoantigen, inducing an autoimmune response, or it causes the pro-inflammatory immune response by activating the UPR (197–200). The IL-17 and IL-23 cytokines including IL-23R are highly activated during HLA-B27 misfolding and UPR activation (198, 201). The production of these cytokines during HLA-B27-induced UPR is mainly contributed from the IRE1α/XBP1s pathway, which enhances IL-23 production during ER stress (202, 203). In addition, IFN-α, IFN-β, or TNFα stimulation in HLA-B27 overexpressing transgenic rats enhanced the BiP and spliced *XBP1* compared to wild-type rats (201, 204). Furthermore, in severe UPR, IRE1α-mediated cell apoptosis would also contribute in the autoimmune response, as apoptotic cells that contain self-antigens known to act as neoantigens induce autoantibody production (205, 206).

In the second mechanism, the ER chaperone protein GRP78 can act as an autoantigen and also known to evoke autoreactive response of B and T-cells (49). IRE1α/XBP1s may contribute to this process through increasing the GRP78 level of expression during ER stress (207). In the case of rheumatoid arthritis (RA), high expression of BiP in the synovium selectively triggered synovial T cells (208, 209). In support to this, autoantibodies of GRP78 were found in 80% of patients with RA (210).

In the third mechanism, defects in the immune tolerance mechanism in IECs may contribute to the progress of inflammatory colitis (211). IRE1β is highly expressed in these cells; interestingly in a study of IRE1β^–/–^ mice, BiP and XBP1s expression levels were increased in IECs indicating ER stress activation (63, 101). In addition, IRE1β^−/−^ mice with dextran sodium sulfate-induced colitis in showed intestinal inflammation earlier than in control mice (62). This is due to the increased permeability of IECs in IRE1β^−/−^ mice which exacerbated their colitis.

The fourth mechanism, may involve increasing the viability of autoreactive cells through the ERAD pathway, enhancing the autoimmune response during ER stress; this is known to reduce the misfolded proteins, easing the ER folding process, and reducing the apoptosis (212–214). The IRE1α/XBP1s pathway increases the expression of several ERAD-associated proteins and contributes to the establishment of homeostasis, further enhancing survival. One of the best examples of this mechanism is synoviolin (SYvN1) in R. IRE1α/XBP1s upregulates ERAD genes and genes important for protein folding such as EDEM1 and protein disulfide isomerase-P5 (81, 215). In addition, ERAD-associated proteins such as EDEM and homocysteine-induced ER protein (HERP) are modified and behave as neoantigens during overloading of the ER (47). This could be one of the contributions in β cell ER stress-mediated neoantigens.

The fifth mechanism is through regulating antigen presentation and affecting Th-1 cell function. The main immune activity in nucleated cells to combat pathogens or tumor cell progression is antigen presentation. Disturbance in antigen presentation may lead to the development of autoimmune disease (216). ER has a key function in MHC class I antigen presentation: usually, peptides derived from pathogens or other antigenic peptides through processing by proteasome will be transported to the ER, where they bind molecules of MHC class I, which exhibit them on the surface of cytotoxic T lymphocytes or CD8^+^ T-cells for the surveillance (217, 218). This binding to MHC class I molecules is required for the functioning of ER proteins, tapasin, and TAP (189, 190, 219). IRE1α activity affects this process during stress by decreasing TAP protein. IRE1α-mediated XBP1s increase the expression of miR-346, which directly represses the translation of TAP and other MHC class I-related mRNAs (189, 190, 192). Subsequently, the reduction of TAP protein leads to defects in MHC class I complex formation and thereby reduces antigen presentation. Interestingly, miR-346 increases the autoimmune response by regulating IL-8 release from activated synoviocytes in RA (220), and in addition, high expression of miR-326 is found in patients with T1D and ongoing islet autoimmunity (221). In addition, RIDD activity is also implicated in the reduction of MHC class I antigen presentation in CD8^+^ cDCs. IRE1α cleaves the crucial MHC class I machinery component, TAPBP mRNA, and reduces the antigen presentation (139), which results in autoimmunity. In support to this mechanism, a report showed that the reduced expression of MHC class I molecules on lymphocytes/lymphoid cells resulted in autoimmunity (222). Furthermore, in another study, the MHC class I molecules of abnormal expression on antigen-presenting cells resulted in the activation of autoreactive T-cells (223). These studies suggest that IRE1α activities interfere with the appropriate development of MHC class I molecules, which are necessarily required for self-immune tolerance; this also enhances the chances of development of autoimmune diseases.

In addition, IRE1α is implicated in increased IFN-γ release from Th-1 cells, which is a hallmark in the pathogenesis of atherosclerosis through IL-18 and IL-1β production from activated NLRP3 inflammasomes (145). The cytokines IL-1β and IL-18 play an important role in the polarization of Th-1 responses (182). Infiltration of these IFN-γ-producing Th-1 cells has been implicated in the development of autoimmune activity in mouse disease models of atherosclerosis (181), multiple sclerosis (224), and T1D as well as in human T1D (225).

## IRE1α Involvement in Autoimmune and Inflammatory Diseases

A faulty immune response can lead to the self-destruction of healthy cells or tissues, causing the development of autoimmune disorders (226). Immune cells target the modified self-cellular components as antigens and induce severe inflammatory responses, which normally lead to cell death (18, 227). There are a number of causative agents, such as oxidative stress; metabolic disorders; genetic factors; ER stress; dysregulation of production of cytokines, such as ILs, IFNs, and TNFα; and auto reactive T-cells; all of these are the hallmarks of autoimmune diseases (193, 226). However, in this section, we highlight the possible mechanisms of IRE1α’s contribution to the progression of autoimmune diseases such as T1D, RA, inflammatory bowel disease (IBD), vitiligo, systemic lupus erythematosus (SLE), and scleroderma (Table 2).

**Table 2:** Possible mechanisms of IRE1α involved in different autoimmune diseases.

Type of disease	IRE1α downstream mechanisms	Reference
Type1 diabetes	IRE1α-associated β cell damage occurs through activation of intrinsic apoptotic pathways Increases insulitis through infiltered immune cells and proinflammatory genes through c-Jun N-terminal kinase (JNK)-AP1 and NFκB pathways β cell death through IRE1α/JNK/CCAAT-enhancer-binding protein homologous protein (CHOP)/DR5 and caspase 12 activation regulated IRE1α-dependent decay (RIDD)-mediated insulitis and β cell death through IL-1β and caspase-1 and caspase-2 activation Enhanced reactive oxygen species (ROS) and NO production through IRE1α/RIDD/thioredoxin-interacting protein (TXNIP) Potentiation of neoantigens development in β cells	(152–158)
Rheumatoid arthritis	Enhances proinflammatory cytokines, such as IL-β, IL-6, and TNFα in infiltered macrophages as well as in fibroblast-like synoviocytes IRE1α contributes increased inflammation and angiogenesis through toll-like receptors mediated activation in infiltered macrophages Enhances synovial fibroblasts survival through endoplasmic reticulum-associated degradation genes upregulation	(87, 88, 144, 159–161)
Systemic lupus erythematosus	Contributes to tissue apoptosis through IRE1α/XBP1s/CHOP and IRE1α/JNK/Bcl-2-associated X protein pathways	(162, 163)
Vitiligo	Cause melanocyte loss through IRE1α/XBP1s-mediated cytokines production IRE1α/XBP1s/TNFα pathway inhibits melanocyte stem cell differentiation Enhances the recruitment of CD8^+^ T cells to skin lesions through increasing the levels of chemokines such as CXCL16 Contributes in increased survival of CD8 α^+^ cDCs Contributes in ROS production at skin lesions through RIDD-mediated TXNIP and nucleotide-binding domain, leucine-rich-containing family, and pyrin domain-containing-3 inflammasome activation	(128, 139, 164–166)
Inflammatory bowel disease	In inflammatory bowel disease, IRE1α contributes to secondary consequences of the disease by inducing the JNK and NFκB-mediated cytokines productions	(167–169)
Systemic sclerosis (scleroderma)	Activated IRE1α/XBP1s pathway in myofibroblasts contributes ER biogenesis, which helps in adaptation to increased protein folding requirement in myofibroblasts IRE1α/XBP1s pathway may also contribute in efficient protein folding by providing ER chaperones, such as glucose-regulated protein 78 IRE1α/RIDD pathway degrades miRNA-150, a repressor αSMA and collagen I and IV expression, which results in enhanced fibrosis IRE1α/JNK/AP1 and IRE1α/NFκB pathways possibly involved endothelin-1 expression in systemic sclerosis	(170, 171)

### Type 1 Diabetes

Type 1 diabetes is a chronic autoimmune disorder characterized by reduced insulin levels and increased blood glucose/hyperglycemia due to autoantigen-induced destruction of pancreatic islet β cells. Subsequently, the burden on the surviving β cells increases (228, 229), which augments the pathologic state of the T1D, which may be due to the ER stress-mediated inflammatory response and also the infiltration of autoreactive immune cells (230). Normally, β cell loss occurs due to impairment in the PTM of endogenous proteins and due to the production of pro-inflammatory cytokines by infiltrated immune cells (41, 231, 232).

Although genetic weakness is a major critical risk factor for β cell destruction (233, 234) and other inflammation inducing environmental factors, such as age, viral infection, drug exposure, ROS, and metabolism fuel the onset or progression of T1D (10). In addition to these environmental triggers, the β cells inherent secretory function even in physiological conditions predisposes them to significantly higher levels of ER stress and UPR activation, compared to other nonsecretory cells (18, 235). Many factors such as pro-inflammatory cytokines, high glucose, and free fatty acids are known to expose β cells to ER stress in Ref. (236–238). Furthermore, these factors induce changes in β cell identity, which makes β cells more vulnerable to autoimmune destruction and results in the progression of T1D (239). Notably, the expression levels of ER stress markers CHOP and BiP are higher in pancreatic islets from T1D individuals compared to healthy individuals (240, 241). In addition, the ER stress-induced misfolded insulin complex can function as a neoantigen and is recognized by autoreactive T-cells (242). Therefore, high level of ER stress would be a common factor that precedes the pathogenesis of T1D (18, 50). These facts signify the association of ER stress in the occurrence of T1D.

β cells express high levels of IRE1α, which is necessary for pro-insulin synthesis (243, 244). In one study, specific deletion of IRE1α in β cells impaired proliferation, proinsulin synthesis processing, and secretion (245). However, in other studies, prolonged activation of the IRE1α pathway in chronic exposure to hyperglycemia triggered alternative molecular pathways along with XBP1s, which led to β cell dysfunction and apoptosis (99, 246, 247). In T1D, IRE1α-associated β cell damage can occur through two processes. First, prolonged ER stress in the β cells due to their high levels of secretion, can induce the intrinsic apoptotic pathway by increasing pro-apoptotic and inflammatory molecules (152, 153). A second process is mediated by insulitis: during the initial phases of the disease immune cells, such as macrophages, DCs, T-cells, and natural killer (NK) cells infiltrate the islets and release pro-inflammatory cytokines, such as IL-1β, TNFα, IFN-γ, IL-17, IL-23, IL-24, and also free radicals ROS and nitric oxide (144, 152, 248). These cytokines enhance β cell apoptosis in T1D (158, 249) by inducing ER stress-mediated activation of AP-1, NFκB, XBP1s, and JNK (54, 238). In addition, pro-inflammatory cytokines stimulate β cells to secrete cytokines and chemokines. These attract T-cells, which then infiltrate the islets, which causes β cell destruction in T1D (152, 153).

IRE1α activates the JNK-AP1 and NFκB pathways, which increases the expression of the pro-inflammatory genes, such as IL-β, TNFα, and IL-6 and regulate the transition from adaptive UPR to apoptotic β-cell death during diabetes (144, 248). Additionally, IRE1α-mediated JNK activation upregulates CHOP and causes β cell death through IRE1α/JNK/CHOP/DR5 expression (154, 155). In the non-obese diabetic mouse (NOD) mice study, the expression of NFκB target genes and ER stress markers increased before the development of hyperglycemia (16), which indicates the inflammatory-mediated IRE1α contribution in T1D. In T1D disorder, pro-inflammatory cytokines are initial mediators of β cell apoptosis (250). Furthermore, the IRE1α/XBP1s pathway also increases NFκB activation by increasing the proteasome-mediated degradation of Forkhead box O1 (FoxO1), an inhibitor of NFκB (Figure 2) (249, 251, 252). In contrast, Hassler et al. recently demonstrated that the IRE1α/XBP1s absence in the islets of adult mouse caused the increase of IL-1β, iNOS, and chemokine (C-X-C motif) ligand 2 (CXCL2) after treatment with high glucose (244). These studies demonstrate that the inflammatory effects of XBP1s are differentially regulated and may depend on the stress intensity. IRE1α also enhances IL-23 expression in DCs (203). This causes the massive T-cell infiltration within the islets and ultimately results in β cell destruction. Furthermore, in a rat model of virus-induced T1D, IRE1α specifically activated caspase-12 and caused β cell death and thus contributed to virus-induced autoimmune T1D (253).

In addition to JNK and NFκB activation, RIDD activity also propagates insulitis and diabetes-related β cell death. IRE1α/RIDD mediates cytokine IL-1β production, and caspase-1 and -2 activation through TXNIP, which is activated by the NLRP3 inflammasome by degradation of TXNIP repressor miRNA (128), and this could also integrate mitochondria-mediated inflammation (129). TXNIP is known to be associated with progression of T1D and is one of the genes, which upregulated during conditions of hyperglycemia in case of human islet cells as well as in animal models of diabetes (156, 157). However, islets from NLRP3^−/−^ and caspase-1^−/−^ mice were not protected from ER stress or high glucose-induced death (254), and knocking out of the NLRP3 inflammasome in Akita mice also did not show the protection to ER stress-induced diabetes progression or β-cell damage (255). These studies indicate three possibilities: first, that the NLRP3 inflammasome is dispensable for β cell death; second that it may be an intermediate molecule in cytokine production; and third, that its role could be context dependent, because elimination of NLRP3 protects against obesity-induced pancreatic damage (256).

Furthermore, IRE1α may potentiate the development of neoantigens in β cells. Exposure of pancreatic β cells to the pro-inflammatory cytokines IL-1β, TNFα, and IFNs induce ER calcium depletion (257). This depletion results in abnormal PTMs through the Ca^2+^-dependent PTM enzyme Tgase2 (18). Furthermore, abnormally folded self-peptides act as neoantigens and increase β cell immunogenicity. These peptides are recognized by autoreactive T-cells, which then destroy the β cells, furthering the pathogenesis of T1D (18). Human islets and human EndoC-βH1 cells exposed to IFN-α showed impaired insulin production *via* increased ER stress and increased XBP1s levels (158). Furthermore, predisposition of pancreatic β cells to ER stress in cases of insulin resistance and obesity exacerbates the activation of IL-1β, TNFα, and NFκB (252). Therefore, the initial activation of IRE1α by mild ER stress exposes β cells to a feedback loop of exacerbated inflammatory responses, causing β cell death and subsequent T1D. These studies suggest that the tight regulation of IRE1α activation, in β cells, is crucial for maintenance of their function. Therefore, a better understanding of IRE1α’s possible role in T1D would open the gate for the discovery of therapeutic options.

### Rheumatoid Arthritis

Rheumatoid arthritis is a chronic autoimmune disorder, in which immune system attacks own body’s tissue and cause bone and joints deformities. This disorder is commonly defined by increased synovial cell proliferation, inflammatory cell infiltration, angiogenesis, and damage in the lining of joints (258). Fibroblast-like synoviocytes (FLS) are key effector cells: they release cytokines and proteases that contribute to cartilage damage (259). The pro-inflammatory cytokines TNFα and IL-1β are significantly upregulated in RA. Synovial fibroblasts in RA are resistant to apoptosis (260), but the mechanisms for this are not yet clear. Recent studies have shown the possible involvement of ER stress due to the increase in the ER stress marker GRP78 in synoviocytes. GRP78 has been implicated in the pathogenesis of RA synovium and synovial cells because of its contribution to synoviocyte proliferation and to angiogenesis, and it can also act as an autoantigen (17). In mice with *Grp78* haploinsufficiency induction of arthritis was suppressed, but GRP78 injection failed to induce arthritis in several strains of rats and mice (210). Additionally, ER stress is well recognized for its functions in cell survival and pro-inflammatory properties; these effects are usually mediated through IRE1α, PERK, and ATF6 (79). Therefore, IRE1α plays a vital role in cell survival, apoptosis, cytokines production, and angiogenesis. Understanding the role of IRE1α in synoviocyte antiapoptotic mechanisms and cytokines production might aid in the design of possible treatment strategies.

Cell surface TLR2 and TLR4, and endosome TLR7, play a very important role in RA pathogenesis by increasing the inflammation and angiogenesis (261). The mechanism through which toll-like receptors (TLRs) induce pathogenesis may be dependent on the IRE1α/XBP1s signaling pathway. The activation of IRE1α/XBP1s signaling found in cells of synovial fluids, obtained from patients with RA, can be due to the TLRs in macrophages, which are usually significantly well expressed in synovial fibroblasts of RA patients (160, 161). In addition, TLRs specifically activated the IRE1α/XBP1s pathway and this is found to be essential for the optimal production of pro-inflammatory cytokines IL-1β, IL-6, and TNFα in macrophages as well as in FLS (144, 159) (Figure 3). In activated synovial fibroblasts of RA patients, XBP1s is highly expressed, but this expression appears to occur independently of ER stress. Instead it is activated through TLR2 and TLR4 ligation-induced IRE1α/XBP1s, leading to increased production of the pro-inflammatory cytokines TNFα and IL-6 (144, 159). This activity of IRE1α could have a special importance in pathogenesis of RA, because expression of TLR2 and TLR4 is distinctly high in the joints of patients with RA (262). Additionally, TLR7 is also implicated in XBP1 induction and IFNα production (179).

Toll-like receptors induced neovascularization in RA is also may be mediated through IRE1α/XBP1s pathway, which is known to upregulate the proangiogenic VEGF-A, IL-1β, IL-6, and IL-8 factors (263). This interrelation of TLRs and IRE1α in RA can further potentiate inflammation *via* increased leukocyte infiltration. Although TLRs are primarily for pathogen receptor recognition, they are also reported as sensing endogenous ligands, such as SNARE associated protein (SNAPIN) (264). In addition, the enhanced TLR2 ligand expression in synovial tissue macrophages has been found to have the importance in the pathogenesis of RA through SNAPIN (265). Therefore, by interference with TLR/IRE1α/XBP1s could be treatment strategy for RA.

Corroboration to above studies, in mice with experimental arthritis, deletion of the IRE1α gene specifically in myeloid tissue- or inhibition of IRE1α with 8-formyl-7-hydroxy-4-methylcoumarin (4μ8c) compound decreased the production of pro-inflammatory cytokines, which further subsidized the joint inflammation (266). This indicates that IRE1α/XBP1s signaling act as a focal point, where different stimuli are converge and function to maintain the activation of FLS. On the other hand, an earlier study reported that the reduction of IRE1α protected the FLS from apoptosis, which led to the enhanced proliferation of synovial cells and influenced the development of RA (267). Furthermore, a recent study reported that hyper-activation of IRE1α inhibited IL-4 and IFN-γ through reducing the *t-bet* and *gata-3* mRNA by its RIDD activity in palmitic acid-treated NK T-cells, thereby suppressing arthritis (268). These studies suggest that FLS in RA maintain an optimal level of IRE1α activation for survival, allowing XBP1s activity but avoiding hyperactivation of IRE1α-mediated apoptosis by promoting synoviolin1 (SYvN1)-associated IRE1α degradation. The pathogenesis of RA is mostly due to the activated synovial fibroblasts, showing enhanced survival and a destructive phenotype (269). This enhanced survival is thought to result from the dysregulation of UPR and ERAD (212, 270, 271). The IRE1α/XBP1s signaling pathway upregulates the ERAD genes during ER stress and may promote the synovial fibroblasts in RA (81, 86–88).

### SLE or Lupus

Systemic lupus erythematosus is an autoimmune disease and it has been described by abnormal apoptosis of healthy tissue in multiple organs of the body, such as lung, heart, skin, kidney, and many other parts (272). SLE pathogenesis is a complex of genetic and environmental factors resulting in an overactive innate immune system, cytokine imbalance, autoantibody production, and abnormal B-cell and T-cell function (273). However, the exact cause of pathogenesis is still not clear. Recently, several studies have tried to establish how ER stress is involved because of its association with other autoimmune diseases, as well as with B-cell, and cytokine production. Additionally, HERP, an ER stress-associated protein has been observed as an autoantigen for anti-DNA antibodies in SLE (274). Further characterization of ER stress-related genes in patients with SLE demonstrated increased expression of XBP1s and CHOP (162). Bone-marrow derived mesenchymal stem cell apoptosis in SLE patients was found to be mediated through the IRE1α/JNK/BaX pathway (163). Differentiation of antibody producing B-cells requires the IRE1α/XBP1s axis: B-cell-specific deletion of *XBP1* protected mice from developing a lupus-like disease (275). These studies indicate that abnormalities in the IRE1α/XBP1s axis may contribute to SLE pathogenesis and could be a target for the treatment. However, some more work needs to be carried out to better appreciate the role of IRE1α in SLE pathogenesis.

### Inflammatory Bowel Disease

Inflammatory bowel disease is generally characterized by recurrent, destructive inflammation of the gastrointestinal tract (276) and comprises both Crohn’s disease and ulcerative colitis forms of IBD. It is estimated to affect millions of people worldwide (277). The definite causes of IBD, either inflammatory-mediated or autoimmune-mediated responses, are highly debated (278). However, there is ample evidence regarding the involvement of autoimmunity in IBD pathogenesis (279, 280). Anti-TNFα drug molecules have shown the positive efficacy against IBD; in fact, TNFα is one of the dominant pro-inflammatory cytokines in autoimmune diseases (281, 282). These studies indicate the possible involvement of autoinflammation in IBD. The loss of tolerance to indigenous enteric flora due to genetic or environmental factors, defects in T-cell function, excessive mucosal DCs, and autoantigens results in IBD pathogenesis (278). ER stress in the intestinal epithelial goblet and Paneth secretory cells is another cause of IBD. ER stress has been reported in IBD inflammation, and all three signal transducing wings (ATF6, IRE1, and PERK) of the UPR are activated (283). The IRE1α role in IBD is focused upon here: as mentioned earlier, both human isoforms IRE1α as well as IRE1β are ubiquitously expressed in the epithelium of the gastro-intestine. Furthermore, IRE1α involvement in optimized production of mucin in intestinal goblet cells indicates the requirement of IRE1α function in the goblet cells ER homeostasis (101). The function of IRE1α/XBP1s signaling pathway is necessarily required for the optimal function and survival of intestinal secretory cells, as such cells are more susceptible to ER stress due to their function (284). Additionally, impairment of the IRE1α/XBP1s axis due to stress leads to the secondary consequence of inflammation (167). With conditional *XBP1* gene knockout mice, specifically at epithelium of small and large intestine Paneth cells as well as goblet cells disappeared (92). This deletion further resulted in IRE1α-activated JNK and NFκB-mediated inflammation, leading to development of the features of human IBD. In addition, the supportive blockade of NFκB activation or the genetic deletion of IRE1α in IECs, protected *Xbp1*ΔIEC mice from spontaneous enteritis (92, 168, 169, 284). p-JNK was increased when XBP1-deficient epithelium was exposed to bacterial antigen, flagellin, and TNFα; a major pathogenic cytokine in IBD was increased. Deficiency of IRE1β and XBP1 within the intestinal epithelium caused the spontaneous inflammation that enhanced the susceptibility to colitis during the treatment with dextran sodium sulfate (62, 92, 285). This could be due to another isoform, IRE1α, as it was observed that, in the absence of XBP1, IRE1α activity augmented JNK phosphorylation. Added to this, IRE1β^−/−^ mice showed accumulation of abnormal MUC2 inside the ER of goblet cells (101). These result in the IL-23 and Th-17 cell inflammatory axis-mediated UPR activation and spontaneous ulcerative colitis (286, 287).

Further, IRE1α accumulation in autophagy defective Atg16l1^ΔIEC^ mice increased Crohn’s disease ileitis (285). In contrast, disruption of IRE1α gene in IECs also led to spontaneous colitis, loss of goblet cells, intestinal epithelial barrier function, and IBD in mice (288) but the colitis could have been due to the lack of XBP1s protein. XBP1 splicing is necessary: in a study of patients with ulcerative colitis, decreased XBP1s levels were observed (289). A recent study reported that the use of the XBP1 agonist HLJ2 inhibited inflammation and ameliorate the ulcerative colitis (290). IRE1α, IRE1β, and XBP1 are very much required for the IECs homeostasis maintenance and also have a functional role in defending against IBDs. However, during severe conditions or during damage caused by other factors, the IRE1α pathway may contribute to extend inflammation and death of IECs.

### Vitiligo

Vitiligo is a condition characterized by white patchy areas on the skin, appeared due to the death of pigment producing melanocytes. It affects approximately 1% of the population worldwide (291). It is a multifactorial disorder with a complex pathogenesis. Oxidative stress and autoimmune mechanisms play major roles in the onset and progression, respectively (164, 292). The mechanisms, which are involved in the triggering of the disease and the spread of lesions, still need to be clarified. However, increased expression of local and systemic cytokines, oxidative stress, and expansion of the ER in melanocytes at the margins of lesions in vitiligo patients indicates the possible involvement of ER stress in pathogenesis (293). Treating melanocytes with chemical inducers of vitiligo, upregulated the expression of XBP1s, and its activation enhanced the release of IL-6 and IL-8 (164). Additionally, polymorphism in the *XBP1*gene increases the risk of developing vitiligo (294). Thus, IRE1α/XBP1s activity in melanocytes contributes to cytokine-associated immune reactions and also melanocyte loss following the onset of vitiligo due to environmental stressors or ROS. TNFα released through IRE1α/XBP1s may inhibit melanocyte stem cell differentiation (165).

Furthermore, CD8^+^ T-cells are key effectors of melanocyte destruction in patients with vitiligo (295, 296). The recruitment of CD8^+^ T-cells to skin lesions is carried out by the IFN-γ-mediated T-cell chemokine receptor, C-X-C motif chemokine receptor 3 (CXCR3), and its ligands CXCL9, CXCL10, and CXCL11, which are abundant in skin biopsy specimens from patients with vitiligo (297). Blockade of this pathway ameliorated the vitiligo in mice as well as in human subjects (298, 299). IRE1α/XBP1s signaling in stressed keratinocytes increased the level of CXCL16, a major chemokine involved in CD8^+^ T-cell recruitment (166). Additionally, IRE1α/XBP1s also contributes to homeostasis and survival of CD8α^+^ conventional DCs (139). Chemically induced skin inflammation in a mouse model showed activation of NLRP3 inflammasome and the downstream effector IL-1β in the milieu of vitiligo (300). IRE1α activity also may lead to activation of NLRP3 inflammasome and release of ROS through regulation of TXNIP expression (128). All these data show the possible involvement of ER stress-induced IRE1α in pathogenesis of vitiligo. However, more studies in relation to IRE1α and the expression patterns of its downstream molecules in clinical samples of vitiligo are necessary for treatment development.

### Systemic Sclerosis (Scleroderma)

IRE1α involvement has also been observed in systemic sclerosis, a complex connective tissue autoimmune diseases, characterized by multi-organ fibrosis due to the fibroblast dysfunction resulted in increased collagen and other extracellular matrix components accumulation in skin and internal organs (301, 302). Increased expression of IRE1α-mediated GRP78 and XBP1s were observed in a subtype of systemic sclerosis, pulmonary arterial hypertension (170). Furthermore, activation of IRE1α contributes to systemic sclerosis through both RIDD and XBP1 splicing activities. RIDD activity degrades the miRNA-150, a repressor of fibrosis components αSMA, collagen I and IV, which influences the myofibroblast formation (171). Spliced XBP1 helps the myofibroblasts in the ER biogenesis and enlargement (303). This activity is required for the myofibroblasts during extracellular matrix proteins secretion and increased ER volume functions as an adaptive mechanism for increased protein folding requirement (171, 303). In the same study, it was shown that, inhibition of IRE1α with 4μ8C prevented the TGF-β induced myofibroblast activation and reduced the fibrosis of liver and skin in animal models. Interestingly, inhibition of IRE1α reverted the diseased phenotypes of myofibroblasts isolated from patients with scleroderma (171). Therefore, targeting the IRE1α with the inhibitors such as 4μ8C could be a possible treatment approach for patients with systemic sclerosis. In addition, endothelin-1 plays a very important functional role in progression of systemic sclerosis (304, 305). This endothelin-1 expression is also regulated by JNK/AP1 and NFκB pathways (306–308). As mentioned above, IRE1α activation increases the JNK/AP1 and NFκB pathways-mediated transcription. Therefore, it could be possible that IRE1α-mediated endothelin-1 expression has a role in systemic sclerosis pathogenesis.

### IRE1α in Other Autoimmune Diseases

In addition, IRE1α importance can be expected in other autoimmune disorders including Sjögren’s syndrome (SS). In this disease, secretory cells are the main type affected, which leads to reduced secretion, resulting in pathologies such as dry mouth and dry skin (309). Normally, salivary gland acinar cells, due to their secretory function, are highly susceptible to ER stress under physiological circumstances due to their high rate of protein synthesis (310–312). Therefore, activation of IRE1α and other UPR molecules is expected due to their regulation of secretion and also to alleviate the ER folding load (87, 313). Further, in patients with SS, accumulation of mucins caused dilatation of the ER, and high levels of pro-inflammatory cytokines were observed in SS patients (214). However, there are fewer data available regarding the IRE1α association with SS. Future studies aiming to characterize the role of IRE1α and its downstream molecules in SS would pave the way for understanding the causes.

IRE1α influences can also be surmised in myasthenia gravis, an autoimmune disease of the neuromuscular junction characterized by muscle fatigue (314). Recently, ER stress has also been implicated in myasthenia gravis, due to the increased expression of ER chaperons GRP78 and GRP94 in skeletal muscle from myasthenia gravis (315, 316). IRE1α/XBP1s, as a potential pathway in ER stress-mediated GRP78 and GRP94 expression (317), possibly have a role in the pathogenesis of myasthenia gravis; however, there is no direct evidence of IRE1α pathway yet.

Furthermore, IRE1α-induced apoptosis has been suspected in the thyroid cytotoxicity that is induced by excessive iodide and fluoride. High levels of IRE1α and XBP1s were observed in the Nthy-ori 3-1 thyroid cell line upon exposure to iodide and fluoride (318). Interestingly, ER stress activation reduced the expression of genes involved in thyroid hormone synthesis. FRTL-5 thyrocytes treated with tunicamycin, an ER stress inducer, showed increased levels of XBP1 and other UPR molecules but also showed a reduction in thyroid hormone synthesis. This indicates the role of ER stress-activated molecules in thyroid hormone synthesis (319). However, IRE1α has not been studied extensively in this disease.

## Modulation of IRE1α Activities in Autoimmune Disease Treatment

The different magnitudes of IRE1α activity under physiological and pathological conditions suggest that the activity levels of its downstream substrates XBP1, ER localized mRNA, miRNA, JNK, and NFκB are crucially dependent on the stress intensity, tissue type, and attributes of the pathology. Interestingly, the structure–activity relationship studies have demonstrated that an allosteric association within the two enzymatic kinase and RDs of IRE1α, which provided the opportunity to modulate its downstream activities (99, 320, 321). Kinase inhibitors/ATP-competitive molecules have been studied to examine how they modulate the RNase activity of IRE1α. Type 1 kinase inhibitors, such as 1NM-PP1, APY29, staurosporine, and sunitinib inhibit autophosphorylation but induce change to the active conformation in both kinase and RDs. Type II kinase inhibitors are kinase-inhibiting RNase attenuators, these allosterically block both kinase and RNase activity by disrupting oligomers of IRE1α (322). Since IRE1α plays a role in both adaptive pro-survival and pro-apoptotic activity, modulating it through inhibition or activation will yield different clinical benefits depending on the type and state of the disease. Many studies have been reported on the application of small chemical modulators in other disease, such as cancer (145, 323, 324). Inhibition of IRE1α with optimized application of KIRA, KIRA6, in rat promoted the cell viability and protected photoreceptor cells function under ER stress (196). Additionally, KIRA6 application in Akita diabetic mice protected pancreatic β cells through improved insulin production and reduced hyperglycemia (196). The details of various chemical modulators of IRE1α have been reviewed elsewhere (325). Treatment of ApoE knockout mice with STF-083010 and 4μ8C, which are IRE1α-specific inhibitors, reduced the hyperlipidemia-induced immune response and alleviated atherosclerosis. Furthermore, treatment with liraglutide, a glucagon-like peptide 1 analog, protected INS-1 cells, a pancreatic cell line, from thapsigargin-induced ER stress-associated cell apoptosis, mainly by suppressing the PERK and IRE1α pathways (326). Application of resveratrol, protected rats against acute kidney injury through inhibition of IRE1α phosphorylation and IRE1α/NFκB pathway-triggered inflammatory response (327).

However, application of IRE1α inhibitor in few experimental models of autoimmune diseases showed the glimpse of treatment possibilities. Treatment with 4μ8c in a mouse inflammatory arthritis model (266) and systemic sclerosis (171) had suppressed the disease phenotypes. In another recent study, application of imatinib, an anti-neoplastic tyrosine kinase inhibitor, protected non-obese diabetic (NOD) mice from T1D by interfering with the interaction between ABL kinase and IRE1α, resulting in reduced enzyme activity (328). In addition to the above small molecule applications, there are some intrinsic molecules that negatively regulate IRE1α activity during stress. However, these molecule interactions are context dependent. Briefly noted here, BaX inhibitor-1, an antiapoptotic, ER stress inhibition molecule that negatively regulates IRE1α-mediated XBP1 splicing and JNK activation, protects against ER stress-associated cell apoptosis (329, 330). In RA, SYVN1 overexpression inhibits IRE1α-mediated cell death by promoting proteasome degradation. This leads to enhanced survival and overgrowth of synovial cells, which escalates the pathogenicity of synovial cells in RA (267). Ubiquitin D expression in a type 1 diabetic condition influenced by IL-1β and IFN-γ reduced the IRE1α/JNK axis-mediated inflammation in cytokine-exposed β cells (331). However, IRE1α is still under investigation for target-specific drug development. In addition, fortilin, a pro-survival molecule, inhibits both kinase and endoribonuclease activities of IRE1α. Treatment protected mice from ER stress-induced liver failure (332). However, the development of therapeutic strategies for modulating IRE1α is still under investigation. Since this molecule plays a role in both cell survival and death, it is very crucial to consider its transition from the pro-survival to the pro-death pathway in developing new therapeutic modes. Fine-tuning of the above mentioned small molecules and intrinsic modulators of IRE1α will probably pave the way forward.

## Conclusion

Accumulating evidence from a variety of recent studies has demonstrated that ER perturbations affect the folding and PTMs of several proteins that develop as auto/neoantigens. Along with this, increased secretion of pro-inflammatory cytokines contributes to the development and pathogenesis of autoimmune diseases. In most cases, the emerging clues suggest that the activation of IRE1α could play a major role in autoimmune disorders. The *XBP1* splicing activities of IRE1α and its RIDD activity are especially known to contribute to the pro-inflammatory responses in several inflammatory disorders. Additionally, the release of AP-1 and NFκB-mediated pro-inflammatory cytokines augments pathogenesis. Furthermore, TLR-mediated activation of IRE1α/XBP1s in an ER stress-independent manner also contributes to the production of pro-inflammatory cytokines, exacerbating the disease condition. Recent studies on the design of small chemical molecules to modulate IRE1α activity are increasing the detailed understanding of IRE1α mechanisms and also may be of therapeutic benefit. Despite the success achieved with the application of small chemical molecules in experimental T1D and RA, more efforts in this direction would better pave the way to meet future challenges with regards to autoimmune disease treatment.

## Author Contributions

RJ and H-JC conceived the concept of the review. RJ wrote the review. RJ and PP designed and formatted the figures. RJ, PP, KB, H-RK, and H-JC read and edited the review manuscript.

## Conflict of Interest Statement

The authors declare that the research was conducted in the absence of any commercial or financial relationships that could be construed as a potential conflict of interest.

## Abbreviations

ASK1, apoptosis signaling kinase1; Bcl-2, B-cell lymphoma family 2; BiP, immunoglobulin binding protein; BaX, Bcl-2-associated X protein; cDC, conventional dendritic cells; CHOP, CCAAT-enhancer-binding protein homologous protein; EDEM, ER degradation-enhancing α-mannosidase-like protein; ERAD, endoplasmic reticulum-associated degradation; GRP78, glucose-regulated protein 78; HLA-B27, human leukocyte antigen B27; IECs, intestinal epithelial cells; JNK, c-Jun N-terminal kinase; NK, natural killer cells; NLRP3, nucleotide-binding domain, leucine-rich-containing family, pyrin domain-containing-3; PTM, post-translational modification; RIDD, regulated IRE1α-dependent decay; ROS, reactive oxygen species; TAP, transporter associated with antigen processing; TAPBP, TAP binding protein; TRAF2, TNF receptor-associated factor 2; TXNIP, thioredoxin-interacting protein; UPR, unfolded protein response.

## References

[1] Liang Y Meng FY Pan HF Ye DQ. A literature review on the patients with autoimmune diseases following vaccination against infections. Hum Vaccin Immunother (2015) 11(9):2274–80. 10.1080/21645515.2015.1009337 25875802 PMC4635701

[2] Wang L Wang FS Gershwin ME Human autoimmune diseases: a comprehensive update. J Intern Med (2015) 278(4):369–95. 10.1111/joim.12395 26212387

[3] Coronel-Restrepo N Posso-Osorio I Naranjo-Escobar J Tobon GJ. Autoimmune diseases and their relation with immunological, neurological and endocrinological axes. Autoimmun Rev (2017) 16(7):684–92. 10.1016/j.autrev.2017.05.002 28479489

[4] Savioli B Abdulahad WH Brouwer E Kallenberg CGM de Souza AWS. Are cytokines and chemokines suitable biomarkers for Takayasu arteritis? Autoimmun Rev (2017) 16(10):1071–8. 10.1016/j.autrev.2017.07.023 28778711

[5] Bogdanos DP Smyk DS Rigopoulou EI Mytilinaiou MG Heneghan MA Selmi C Twin studies in autoimmune disease: genetics, gender and environment. J Autoimmun (2012) 38(2–3):J156–69. 10.1016/j.jaut.2011.11.003 22177232

[6] Selmi C Leung PS Sherr DH Diaz M Nyland JF Monestier M Mechanisms of environmental influence on human autoimmunity: a National Institute of Environmental Health Sciences expert panel workshop. J Autoimmun (2012) 39(4):272–84. 10.1016/j.jaut.2012.05.007 22749494

[7] Takasu N Asawa T Komiya I Nagasawa Y Yamada T. Alloxan-induced DNA strand breaks in pancreatic islets. Evidence for H2O2 as an intermediate. J Biol Chem (1991) 266(4):2112–4. 1989973

[8] Sosenko JM Palmer JP Rafkin-Mervis L Krischer JP Cuthbertson D Mahon J Incident dysglycemia and progression to type 1 diabetes among participants in the Diabetes Prevention Trial-Type 1. Diabetes Care (2009) 32(9):1603–7. 10.2337/dc08-2140 19487644 PMC2732147

[9] Schulte BM Kramer M Ansems M Lanke KH van Doremalen N Piganelli JD Phagocytosis of enterovirus-infected pancreatic beta-cells triggers innate immune responses in human dendritic cells. Diabetes (2010) 59(5):1182–91. 10.2337/db09-1071 20071599 PMC2857898

[10] Delmastro-Greenwood MM Tse HM Piganelli JD. Effects of metalloporphyrins on reducing inflammation and autoimmunity. Antioxid Redox Signal (2014) 20(15):2465–77. 10.1089/ars.2013.5257 23472672

[11] Morgan NG Leete P Foulis AK Richardson SJ. Islet inflammation in human type 1 diabetes mellitus. IUBMB Life (2014) 66(11):723–34. 10.1002/iub.1330 25504835

[12] Ito A Hong C Oka K Salazar JV Diehl C Witztum JL Cholesterol accumulation in CD11c(+) immune cells is a causal and targetable factor in autoimmune disease. Immunity (2016) 45(6):1311–26. 10.1016/j.immuni.2016.11.008 28002731 PMC5181791

[13] Crowe W Allsopp PJ Watson GE Magee PJ Strain JJ Armstrong DJ Mercury as an environmental stimulus in the development of autoimmunity – a systematic review. Autoimmun Rev (2017) 16(1):72–80. 10.1016/j.autrev.2016.09.020 27666813

[14] Chaudhari N Talwar P Parimisetty A Lefebvre d’Hellencourt C Ravanan P. A molecular web: endoplasmic reticulum stress, inflammation, and oxidative stress. Front Cell Neurosci (2014) 8:213. 10.3389/fncel.2014.00213 25120434 PMC4114208

[15] Foufelle F Fromenty B. Role of endoplasmic reticulum stress in drug-induced toxicity. Pharmacol Res Perspect (2016) 4(1):e00211. 10.1002/prp2.211 26977301 PMC4777263

[16] Tersey SA Nishiki Y Templin AT Cabrera SM Stull ND Colvin SC Islet beta-cell endoplasmic reticulum stress precedes the onset of type 1 diabetes in the nonobese diabetic mouse model. Diabetes (2012) 61(4):818–27. 10.2337/db11-1293 22442300 PMC3314371

[17] Park YJ Yoo SA Kim WU. Role of endoplasmic reticulum stress in rheumatoid arthritis pathogenesis. J Korean Med Sci (2014) 29(1):2–11. 10.3346/jkms.2014.29.1.2 24431899 PMC3890471

[18] Marre ML Profozich JL Coneybeer JT Geng X Bertera S Ford MJ Inherent ER stress in pancreatic islet beta cells causes self-recognition by autoreactive T cells in type 1 diabetes. J Autoimmun (2016) 72:33–46. 10.1016/j.jaut.2016.04.009 27173406 PMC4958612

[19] Bettigole SE Glimcher LH Endoplasmic reticulum stress in immunity. Annu Rev Immunol (2015) 33:107–38. 10.1146/annurev-immunol-032414-112116 25493331

[20] Feldmann M Brennan FM Maini RN. Role of cytokines in rheumatoid arthritis. Annu Rev Immunol (1996) 14:397–440. 10.1146/annurev.immunol.14.1.397 8717520

[21] Anelli T Sitia R. Protein quality control in the early secretory pathway. EMBO J (2008) 27(2):315–27. 10.1038/sj.emboj.7601974 18216874 PMC2234347

[22] Rashid HO Yadav RK Kim HR Chae HJ. ER stress: autophagy induction, inhibition and selection. Autophagy (2015) 11(11):1956–77. 10.1080/15548627.2015.1091141 26389781 PMC4824587

[23] Hetz C Saxena S. ER stress and the unfolded protein response in neurodegeneration. Nat Rev Neurol (2017) 13(8):477–91. 10.1038/nrneurol.2017.99 28731040

[24] Balchin D Hayer-Hartl M Hartl FU. In vivo aspects of protein folding and quality control. Science (2016) 353(6294):aac4354. 10.1126/science.aac4354 27365453

[25] Schroder M Kaufman RJ. ER stress and the unfolded protein response. Mutat Res (2005) 569(1–2):29–63. 10.1016/j.mrfmmm.2004.06.056 15603751

[26] Tu BP Weissman JS. Oxidative protein folding in eukaryotes: mechanisms and consequences. J Cell Biol (2004) 164(3):341–6. 10.1083/jcb.200311055 14757749 PMC2172237

[27] Melloul D Marshak S Cerasi E. Regulation of insulin gene transcription. Diabetologia (2002) 45(3):309–26. 10.1007/s00125-001-0728-y 11914736

[28] Papa FR Endoplasmic reticulum stress, pancreatic beta-cell degeneration, and diabetes. Cold Spring Harb Perspect Med (2012) 2(9):a007666 10.1101/cshperspect.a007666 22951443 PMC3426819

[29] Young CL Robinson AS. Protein folding and secretion: mechanistic insights advancing recombinant protein production in *S. cerevisiae*. Curr Opin Biotechnol (2014) 30:168–77. 10.1016/j.copbio.2014.06.018 25032908 PMC4253588

[30] Yoshida H ER stress and diseases. FEBS J (2007) 274(3):630–58. 10.1111/j.1742-4658.2007.05639.x 17288551

[31] Corazzari M Gagliardi M Fimia GM Piacentini M. Endoplasmic reticulum stress, unfolded protein response, and cancer cell fate. Front Oncol (2017) 7:78. 10.3389/fonc.2017.00078 28491820 PMC5405076

[32] Perera N Miller JL Zitzmann N. The role of the unfolded protein response in dengue virus pathogenesis. Cell Microbiol (2017) 19(5):e12734. 10.1111/cmi.12734 28207988

[33] Feldman DE Chauhan V Koong AC. The unfolded protein response: a novel component of the hypoxic stress response in tumors. Mol Cancer Res (2005) 3(11):597–605. 10.1158/1541-7786.MCR-05-0221 16317085

[34] Hetz C Papa FR The unfolded protein response and cell fate control. Mol Cell (2018) 69(2):169–81. 10.1016/j.molcel.2017.06.017 29107536

[35] Wu H Ng BS Thibault G. Endoplasmic reticulum stress response in yeast and humans. Biosci Rep (2014) 34(4):e00118. 10.1042/BSR20140058 24909749 PMC4076835

[36] Walter P Ron D. The unfolded protein response: from stress pathway to homeostatic regulation. Science (2011) 334(6059):1081–6. 10.1126/science.1209038 22116877

[37] Lin JH Walter P Yen TS. Endoplasmic reticulum stress in disease pathogenesis. Annu Rev Pathol (2008) 3:399–425. 10.1146/annurev.pathmechdis.3.121806.151434 18039139 PMC3653419

[38] Kaufman RJ Orchestrating the unfolded protein response in health and disease. J Clin Invest (2002) 110(10):1389–98. 10.1172/JCI0216886 12438434 PMC151822

[39] Lin JH Li H Yasumura D Cohen HR Zhang C Panning B IRE1 signaling affects cell fate during the unfolded protein response. Science (2007) 318(5852):944–9. 10.1126/science.1146361 17991856 PMC3670588

[40] Urano F Wang X Bertolotti A Zhang Y Chung P Harding HP Coupling of stress in the ER to activation of JNK protein kinases by transmembrane protein kinase IRE1. Science (2000) 287(5453):664–6. 10.1126/science.287.5453.664 10650002

[41] Dunne JL Overbergh L Purcell AW Mathieu C. Posttranslational modifications of proteins in type 1 diabetes: the next step in finding the cure? Diabetes (2012) 61(8):1907–14. 10.2337/db11-1675 22826307 PMC3402302

[42] Clark AL Urano F. Endoplasmic reticulum stress in beta cells and autoimmune diabetes. Curr Opin Immunol (2016) 43:60–6. 10.1016/j.coi.2016.09.006 27718448 PMC5125892

[43] Marre ML Piganelli JD Environmental factors contribute to beta cell endoplasmic reticulum stress and neo-antigen formation in type 1 diabetes. Front Endocrinol (2017) 8:262 10.3389/fendo.2017.00262 PMC5626851 29033899

[44] Sherr J Sosenko J Skyler JS Herold KC. Prevention of type 1 diabetes: the time has come. Nat Clin Pract Endocrinol Metab (2008) 4(6):334–43. 10.1038/ncpendmet0832 18446141

[45] Stadinski BD Delong T Reisdorph N Reisdorph R Powell RL Armstrong M Chromogranin A is an autoantigen in type 1 diabetes. Nat Immunol (2010) 11(3):225–31. 10.1038/ni.1844 20139986 PMC3166626

[46] Roep BO Kracht MJ van Lummel M Zaldumbide A. A roadmap of the generation of neoantigens as targets of the immune system in type 1 diabetes. Curr Opin Immunol (2016) 43:67–73. 10.1016/j.coi.2016.09.007 27723537

[47] Morito D Nagata K. ER stress proteins in autoimmune and inflammatory diseases. Front Immunol (2012) 3:48. 10.3389/fimmu.2012.00048 22566930 PMC3342303

[48] Rondas D Crevecoeur I D’Hertog W Ferreira GB Staes A Garg AD Citrullinated glucose-regulated protein 78 is an autoantigen in type 1 diabetes. Diabetes (2015) 64(2):573–86. 10.2337/db14-0621 25204978

[49] Panayi GS Corrigall VM. BiP regulates autoimmune inflammation and tissue damage. Autoimmun Rev (2006) 5(2):140–2. 10.1016/j.autrev.2005.08.006 16431346

[50] Marre ML James EA Piganelli JD beta cell ER stress and the implications for immunogenicity in type 1 diabetes. Front Cell Dev Biol (2015) 3:67 10.3389/fcell.2015.00067 26579520 PMC4621612

[51] Kim S Joe Y Kim HJ Kim YS Jeong SO Pae HO Endoplasmic reticulum stress-induced IRE1alpha activation mediates cross-talk of GSK-3beta and XBP-1 to regulate inflammatory cytokine production. J Immunol (2015) 194(9):4498–506. 10.4049/jimmunol.1401399 25821218 PMC4400814

[52] Moudgil KD Choubey D. Cytokines in autoimmunity: role in induction, regulation, and treatment. J Interferon Cytokine Res (2011) 31(10):695–703. 10.1089/jir.2011.0065 21942420 PMC3189547

[53] Goules AV Tzioufas AG Primary Sjgren’s syndrome: clinical phenotypes, outcome and the development of biomarkers. Autoimmun Rev (2016) 15(7):695–703. 10.1016/j.autrev.2016.03.004 26970487

[54] Brozzi F Nardelli TR Lopes M Millard I Barthson J Igoillo-Esteve M Cytokines induce endoplasmic reticulum stress in human, rat and mouse beta cells via different mechanisms. Diabetologia (2015) 58(10):2307–16. 10.1007/s00125-015-3669-6 26099855

[55] Maurel M Chevet E Tavernier J Gerlo S. Getting RIDD of RNA: IRE1 in cell fate regulation. Trends Biochem Sci (2014) 39(5):245–54. 10.1016/j.tibs.2014.02.008 24657016

[56] Moore K Hollien J. Ire1-mediated decay in mammalian cells relies on mRNA sequence, structure, and translational status. Mol Biol Cell (2015) 26(16):2873–84. 10.1091/mbc.E15-02-0074 26108623 PMC4571326

[57] Ishikawa T Kashima M Nagano AJ Ishikawa-Fujiwara T Kamei Y Todo T Unfolded protein response transducer IRE1-mediated signaling independent of XBP1 mRNA splicing is not required for growth and development of medaka fish. Elife (2017) 6:e26845. 10.7554/eLife.26845 28952924 PMC5636610

[58] Nikawa J Yamashita S. IRE1 encodes a putative protein kinase containing a membrane-spanning domain and is required for inositol phototrophy in *Saccharomyces cerevisiae*. Mol Microbiol (1992) 6(11):1441–6. 10.1111/j.1365-2958.1992.tb00864.x 1625574

[59] Cox JS Shamu CE Walter P. Transcriptional induction of genes encoding endoplasmic reticulum resident proteins requires a transmembrane protein kinase. Cell (1993) 73(6):1197–206. 10.1016/0092-8674(93)90648-A 8513503

[60] Mori K Ma W Gething MJ Sambrook J. A transmembrane protein with a cdc2+/CDC28-related kinase activity is required for signaling from the ER to the nucleus. Cell (1993) 74(4):743–56. 10.1016/0092-8674(93)90521-Q 8358794

[61] Ron D Walter P. Signal integration in the endoplasmic reticulum unfolded protein response. Nat Rev Mol Cell Biol (2007) 8(7):519–29. 10.1038/nrm2199 17565364

[62] Bertolotti A Wang X Novoa I Jungreis R Schlessinger K Cho JH Increased sensitivity to dextran sodium sulfate colitis in IRE1beta-deficient mice. J Clin Invest (2001) 107(5):585–93. 10.1172/JCI11476 11238559 PMC199427

[63] Martino MB Jones L Brighton B Ehre C Abdulah L Davis CW The ER stress transducer IRE1beta is required for airway epithelial mucin production. Mucosal Immunol (2013) 6(3):639–54. 10.1038/mi.2012.105 23168839 PMC4031691

[64] Iwawaki T Hosoda A Okuda T Kamigori Y Nomura-Furuwatari C Kimata Y Translational control by the ER transmembrane kinase/ribonuclease IRE1 under ER stress. Nat Cell Biol (2001) 3(2):158–64. 10.1038/35083107 11175748

[65] Li H Korennykh AV Behrman SL Walter P. Mammalian endoplasmic reticulum stress sensor IRE1 signals by dynamic clustering. Proc Natl Acad Sci U S A (2010) 107(37):16113–8. 10.1073/pnas.1010580107 20798350 PMC2941319

[66] Kimata Y Kimata YI Shimizu Y Abe H Farcasanu IC Takeuchi M Genetic evidence for a role of BiP/Kar2 that regulates Ire1 in response to accumulation of unfolded proteins. Mol Biol Cell (2003) 14(6):2559–69. 10.1091/mbc.e02-11-0708 12808051 PMC194903

[67] Kimata Y Oikawa D Shimizu Y Ishiwata-Kimata Y Kohno K. A role for BiP as an adjustor for the endoplasmic reticulum stress-sensing protein Ire1. J Cell Biol (2004) 167(3):445–56. 10.1083/jcb.200405153 15520230 PMC2172501

[68] Okamura K Kimata Y Higashio H Tsuru A Kohno K. Dissociation of Kar2p/BiP from an ER sensory molecule, Ire1p, triggers the unfolded protein response in yeast. Biochem Biophys Res Commun (2000) 279(2):445–50. 10.1006/bbrc.2000.3987 11118306

[69] Shamu CE Walter P. Oligomerization and phosphorylation of the Ire1p kinase during intracellular signaling from the endoplasmic reticulum to the nucleus. EMBO J (1996) 15(12):3028–39. 8670804 PMC450244

[70] Korennykh AV Egea PF Korostelev AA Finer-Moore J Zhang C Shokat KM The unfolded protein response signals through high-order assembly of Ire1. Nature (2009) 457(7230):687–93. 10.1038/nature07661 19079236 PMC2846394

[71] Pincus D Chevalier MW Aragon T van Anken E Vidal SE El-Samad H BiP binding to the ER-stress sensor Ire1 tunes the homeostatic behavior of the unfolded protein response. PLoS Biol (2010) 8(7):e1000415. 10.1371/journal.pbio.1000415 20625545 PMC2897766

[72] Ali MM Bagratuni T Davenport EL Nowak PR Silva-Santisteban MC Hardcastle A Structure of the Ire1 autophosphorylation complex and implications for the unfolded protein response. EMBO J (2011) 30(5):894–905. 10.1038/emboj.2011.18 21317875 PMC3049214

[73] Gardner BM Pincus D Gotthardt K Gallagher CM Walter P. Endoplasmic reticulum stress sensing in the unfolded protein response. Cold Spring Harb Perspect Biol (2013) 5(3):a013169. 10.1101/cshperspect.a013169 23388626 PMC3578356

[74] Karagoz GE Acosta-Alvear D Nguyen HT Lee CP Chu F Walter P. An unfolded protein-induced conformational switch activates mammalian IRE1. Elife (2017) 6:e30700. 10.7554/eLife.30700 28971800 PMC5699868

[75] Promlek T Ishiwata-Kimata Y Shido M Sakuramoto M Kohno K Kimata Y. Membrane aberrancy and unfolded proteins activate the endoplasmic reticulum stress sensor Ire1 in different ways. Mol Biol Cell (2011) 22(18):3520–32. 10.1091/mbc.E11-04-0295 21775630 PMC3172275

[76] Kitai Y Ariyama H Kono N Oikawa D Iwawaki T Arai H Membrane lipid saturation activates IRE1alpha without inducing clustering. Genes Cells (2013) 18(9):798–809. 10.1111/gtc.12074 23803178

[77] Volmer R van der Ploeg K Ron D. Membrane lipid saturation activates endoplasmic reticulum unfolded protein response transducers through their transmembrane domains. Proc Natl Acad Sci U S A (2013) 110(12):4628–33. 10.1073/pnas.1217611110 23487760 PMC3606975

[78] Uemura A Oku M Mori K Yoshida H. Unconventional splicing of XBP1 mRNA occurs in the cytoplasm during the mammalian unfolded protein response. J Cell Sci (2009) 122(Pt 16):2877–86. 10.1242/jcs.040584 19622636

[79] Hetz C Martinon F Rodriguez D Glimcher LH The unfolded protein response: integrating stress signals through the stress sensor IRE1alpha. Physiol Rev (2011) 91(4):1219–43. 10.1152/physrev.00001.2011 22013210

[80] Jurkin J Henkel T Nielsen AF Minnich M Popow J Kaufmann T The mammalian tRNA ligase complex mediates splicing of XBP1 mRNA and controls antibody secretion in plasma cells. EMBO J (2014) 33(24):2922–36. 10.15252/embj.201490332 25378478 PMC4282640

[81] Calfon M Zeng H Urano F Till JH Hubbard SR Harding HP IRE1 couples endoplasmic reticulum load to secretory capacity by processing the XBP-1 mRNA. Nature (2002) 415(6867):92–6. 10.1038/415092a 11780124

[82] Lu Y Liang FX Wang X. A synthetic biology approach identifies the mammalian UPR RNA ligase RtcB. Mol Cell (2014) 55(5):758–70. 10.1016/j.molcel.2014.06.032 25087875 PMC4156904

[83] Aragon T van Anken E Pincus D Serafimova IM Korennykh AV Rubio CA Messenger RNA targeting to endoplasmic reticulum stress signalling sites. Nature (2009) 457(7230):736–40. 10.1038/nature07641 19079237 PMC2768538

[84] Lee AH Iwakoshi NN Glimcher LH. XBP-1 regulates a subset of endoplasmic reticulum resident chaperone genes in the unfolded protein response. Mol Cell Biol (2003) 23(21):7448–59. 10.1128/MCB.23.21.7448-7459.2003 14559994 PMC207643

[85] Hendershot LM. The ER function BiP is a master regulator of ER function. Mt Sinai J Med (2004) 71(5):289–97. 15543429

[86] Acosta-Alvear D Zhou Y Blais A Tsikitis M Lents NH Arias C XBP1 controls diverse cell type- and condition-specific transcriptional regulatory networks. Mol Cell (2007) 27(1):53–66. 10.1016/j.molcel.2007.06.011 17612490

[87] Moore KA Hollien J. The unfolded protein response in secretory cell function. Annu Rev Genet (2012) 46:165–83. 10.1146/annurev-genet-110711-155644 22934644

[88] Wong MY DiChiara AS Suen PH Chen K Doan ND Shoulders MD Adapting secretory proteostasis and function through the unfolded protein response. Curr Top Microbiol Immunol (2018) 414:1–25. 10.1007/82_2017_56 28929194 PMC5860992

[89] Shaffer AL Shapiro-Shelef M Iwakoshi NN Lee AH Qian SB Zhao H XBP1, downstream of Blimp-1, expands the secretory apparatus and other organelles, and increases protein synthesis in plasma cell differentiation. Immunity (2004) 21(1):81–93. 10.1016/j.immuni.2004.06.010 15345222

[90] Lee AH Chu GC Iwakoshi NN Glimcher LH. XBP-1 is required for biogenesis of cellular secretory machinery of exocrine glands. EMBO J (2005) 24(24):4368–80. 10.1038/sj.emboj.7600903 16362047 PMC1356340

[91] Sriburi R Bommiasamy H Buldak GL Robbins GR Frank M Jackowski S Coordinate regulation of phospholipid biosynthesis and secretory pathway gene expression in XBP-1(S)-induced endoplasmic reticulum biogenesis. J Biol Chem (2007) 282(10):7024–34. 10.1074/jbc.M609490200 17213183

[92] Kaser A Lee AH Franke A Glickman JN Zeissig S Tilg H XBP1 links ER stress to intestinal inflammation and confers genetic risk for human inflammatory bowel disease. Cell (2008) 134(5):743–56. 10.1016/j.cell.2008.07.021 18775308 PMC2586148

[93] Iwawaki T Akai R Yamanaka S Kohno K. Function of IRE1 alpha in the placenta is essential for placental development and embryonic viability. Proc Natl Acad Sci U S A (2009) 106(39):16657–62. 10.1073/pnas.0903775106 19805353 PMC2757843

[94] Hollien J Weissman JS. Decay of endoplasmic reticulum-localized mRNAs during the unfolded protein response. Science (2006) 313(5783):104–7. 10.1126/science.1129631 16825573

[95] Hollien J Lin JH Li H Stevens N Walter P Weissman JS. Regulated Ire1-dependent decay of messenger RNAs in mammalian cells. J Cell Biol (2009) 186(3):323–31. 10.1083/jcb.200903014 19651891 PMC2728407

[96] Bhattacharyya S Sen U Vrati S. Regulated IRE1-dependent decay pathway is activated during Japanese encephalitis virus-induced unfolded protein response and benefits viral replication. J Gen Virol (2014) 95(Pt 1):71–9. 10.1099/vir.0.057265-0 24114795

[97] Fink SL Jayewickreme TR Molony RD Iwawaki T Landis CS Lindenbach BD IRE1alpha promotes viral infection by conferring resistance to apoptosis. Sci Signal (2017) 10(482):eaai7814 10.1126/scisignal.aai7814 28588082 PMC5535312

[98] Lencer WI DeLuca H Grey MJ Cho JA. Innate immunity at mucosal surfaces: the IRE1-RIDD-RIG-I pathway. Trends Immunol (2015) 36(7):401–9. 10.1016/j.it.2015.05.006 26093676 PMC4490948

[99] Han D Lerner AG Vande Walle L Upton JP Xu W Hagen A IRE1alpha kinase activation modes control alternate endoribonuclease outputs to determine divergent cell fates. Cell (2009) 138(3):562–75. 10.1016/j.cell.2009.07.017 19665977 PMC2762408

[100] Bright MD Itzhak DN Wardell CP Morgan GJ Davies FE. Cleavage of BLOC1S1 mRNA by IRE1 is sequence specific, temporally separate from XBP1 splicing, and dispensable for cell viability under acute endoplasmic reticulum stress. Mol Cell Biol (2015) 35(12):2186–202. 10.1128/MCB.00013-15 25870107 PMC4438243

[101] Tsuru A Fujimoto N Takahashi S Saito M Nakamura D Iwano M Negative feedback by IRE1beta optimizes mucin production in goblet cells. Proc Natl Acad Sci U S A (2013) 110(8):2864–9. 10.1073/pnas.1212484110 23386727 PMC3581977

[102] Chen Y Brandizzi F. IRE1: ER stress sensor and cell fate executor. Trends Cell Biol (2013) 23(11):547–55. 10.1016/j.tcb.2013.06.005 23880584 PMC3818365

[103] Hiramatsu N Chiang WC Kurt TD Sigurdson CJ Lin JH. Multiple mechanisms of unfolded protein response-induced cell death. Am J Pathol (2015) 185(7):1800–8. 10.1016/j.ajpath.2015.03.009 25956028 PMC4484218

[104] Coelho DS Domingos PM. Physiological roles of regulated Ire1 dependent decay. Front Genet (2014) 5:76. 10.3389/fgene.2014.00076 24795742 PMC3997004

[105] Wu J He GT Zhang WJ Xu J Huang QB IRE1alpha signaling pathways involved in mammalian cell fate determination. Cell Physiol Biochem (2016) 38(3):847–58. 10.1159/000443039 26910807

[106] Nishitoh H Matsuzawa A Tobiume K Saegusa K Takeda K Inoue K ASK1 is essential for endoplasmic reticulum stress-induced neuronal cell death triggered by expanded polyglutamine repeats. Genes Dev (2002) 16(11):1345–55. 10.1101/gad.992302 12050113 PMC186318

[107] Zeng T Peng L Chao H Xi H Fu B Wang Y IRE1alpha-TRAF2-ASK1 complex-mediated endoplasmic reticulum stress and mitochondrial dysfunction contribute to CXC195-induced apoptosis in human bladder carcinoma T24 cells. Biochem Biophys Res Commun (2015) 460(3):530–6. 10.1016/j.bbrc.2015.03.064 25797626

[108] Zhang J Liang Y Lin Y Liu Y YouYou Yin W IRE1alpha-TRAF2-ASK1 pathway is involved in CSTMP-induced apoptosis and ER stress in human non-small cell lung cancer A549 cells. Biomed Pharmacother (2016) 82:281–9. 10.1016/j.biopha.2016.04.050 27470364

[109] Tournier C Hess P Yang DD Xu J Turner TK Nimnual A Requirement of JNK for stress-induced activation of the cytochrome c-mediated death pathway. Science (2000) 288(5467):870–4. 10.1126/science.288.5467.870 10797012

[110] Lei K Davis RJ. JNK phosphorylation of Bim-related members of the Bcl2 family induces Bax-dependent apoptosis. Proc Natl Acad Sci U S A (2003) 100(5):2432–7. 10.1073/pnas.0438011100 12591950 PMC151358

[111] Gross A McDonnell JM Korsmeyer SJ BCL-2 family members and the mitochondria in apoptosis. Genes Dev (1999) 13(15):1899–911. 10.1101/gad.13.15.1899 10444588

[112] Yamamoto K Ichijo H Korsmeyer SJ. BCL-2 is phosphorylated and inactivated by an ASK1/Jun N-terminal protein kinase pathway normally activated at G(2)/M. Mol Cell Biol (1999) 19(12):8469–78. 10.1128/MCB.19.12.8469 10567572 PMC84954

[113] Wang XT Pei DS Xu J Guan QH Sun YF Liu XM Opposing effects of Bad phosphorylation at two distinct sites by Akt1 and JNK1/2 on ischemic brain injury. Cell Signal (2007) 19(9):1844–56. 10.1016/j.cellsig.2007.04.005 17555943

[114] Win S Than TA Fernandez-Checa JC Kaplowitz N. JNK interaction with Sab mediates ER stress induced inhibition of mitochondrial respiration and cell death. Cell Death Dis (2014) 5:e989. 10.1038/cddis.2013.522 24407242 PMC4040675

[115] Jochum W Passegue E Wagner EF. AP-1 in mouse development and tumorigenesis. Oncogene (2001) 20(19):2401–12. 10.1038/sj.onc.1204389 11402336

[116] Dhanasekaran DN Reddy EP. JNK signaling in apoptosis. Oncogene (2008) 27(48):6245–51. 10.1038/onc.2008.301 18931691 PMC3063296

[117] Zheng GF Cai Z Meng XK Zhang Y Zhu W Pang XY Unfolded protein response mediated JNK/AP-1 signal transduction, a target for ovarian cancer treatment. Int J Clin Exp Pathol (2015) 8(6):6505–11. 26261528 PMC4525862

[118] Nakagawa T Zhu H Morishima N Li E Xu J Yankner BA Caspase-12 mediates endoplasmic-reticulum-specific apoptosis and cytotoxicity by amyloid-beta. Nature (2000) 403(6765):98–103. 10.1038/47513 10638761

[119] Yoneda T Imaizumi K Oono K Yui D Gomi F Katayama T Activation of caspase-12, an endoplastic reticulum (ER) resident caspase, through tumor necrosis factor receptor-associated factor 2-dependent mechanism in response to the ER stress. J Biol Chem (2001) 276(17):13935–40. 10.1074/jbc.M010677200 11278723

[120] Yang Q Kim YS Lin Y Lewis J Neckers L Liu ZG. Tumour necrosis factor receptor 1 mediates endoplasmic reticulum stress-induced activation of the MAP kinase JNK. EMBO Rep (2006) 7(6):622–7. 10.1038/sj.embor.7400687 16680093 PMC1479600

[121] Estornes Y Aguileta MA Dubuisson C De Keyser J Goossens V Kersse K RIPK1 promotes death receptor-independent caspase-8-mediated apoptosis under unresolved ER stress conditions. Cell Death Dis (2015) 6:e1798 10.1038/cddis.2015.175 26111060 PMC4669845

[122] Kaneko M Niinuma Y Nomura Y. Activation signal of nuclear factor-kappa B in response to endoplasmic reticulum stress is transduced via IRE1 and tumor necrosis factor receptor-associated factor 2. Biol Pharm Bull (2003) 26(7):931–5. 10.1248/bpb.26.931 12843613

[123] Hamid T Guo SZ Kingery JR Xiang X Dawn B Prabhu SD Cardiomyocyte NF-kappaB p65 promotes adverse remodelling, apoptosis, and endoplasmic reticulum stress in heart failure. Cardiovasc Res (2011) 89(1):129–38. 10.1093/cvr/cvq274 20797985 PMC3002872

[124] Han CY Lim SW Koo JH Kim W Kim SG. PHLDA3 overexpression in hepatocytes by endoplasmic reticulum stress via IRE1-Xbp1s pathway expedites liver injury. Gut (2016) 65(8):1377–88. 10.1136/gutjnl-2014-308506 25966993 PMC4975835

[125] Li Y Guo Y Tang J Jiang J Chen Z. New insights into the roles of CHOP-induced apoptosis in ER stress. Acta Biochim Biophys Sin (Shanghai) (2014) 46(8):629–40. 10.1093/abbs/gmu048 25016584

[126] Upton JP Austgen K Nishino M Coakley KM Hagen A Han D caspase-2 cleavage of BID is a critical apoptotic signal downstream of endoplasmic reticulum stress. Mol Cell Biol (2008) 28(12):3943–51. 10.1128/MCB.00013-08 18426910 PMC2423129

[127] Upton JP Wang L Han D Wang ES Huskey NE Lim L IRE1alpha cleaves select microRNAs during ER stress to derepress translation of proapoptotic caspase-2. Science (2012) 338(6108):818–22. 10.1126/science.1226191 23042294 PMC3742121

[128] Lerner AG Upton JP Praveen PV Ghosh R Nakagawa Y Igbaria A IRE1alpha induces thioredoxin-interacting protein to activate the NLRP3 inflammasome and promote programmed cell death under irremediable ER stress. Cell Metab (2012) 16(2):250–64. 10.1016/j.cmet.2012.07.007 22883233 PMC4014071

[129] Bronner DN Abuaita BH Chen X Fitzgerald KA Nunez G He Y Endoplasmic reticulum stress activates the inflammasome via NLRP3- and caspase-2-driven mitochondrial damage. Immunity (2015) 43(3):451–62. 10.1016/j.immuni.2015.08.008 26341399 PMC4582788

[130] Janssens S Pulendran B Lambrecht BN. Emerging functions of the unfolded protein response in immunity. Nat Immunol (2014) 15(10):910–9. 10.1038/ni.2991 25232821 PMC4388558

[131] Grootjans J Kaser A Kaufman RJ Blumberg RS. The unfolded protein response in immunity and inflammation. Nat Rev Immunol (2016) 16(8):469–84. 10.1038/nri.2016.62 27346803 PMC5310224

[132] Rutkowski DT Hegde RS. Regulation of basal cellular physiology by the homeostatic unfolded protein response. J Cell Biol (2010) 189(5):783–94. 10.1083/jcb.201003138 20513765 PMC2878945

[133] Reimold AM Iwakoshi NN Manis J Vallabhajosyula P Szomolanyi-Tsuda E Gravallese EM Plasma cell differentiation requires the transcription factor XBP-1. Nature (2001) 412(6844):300–7. 10.1038/35085509 11460154

[134] Iwakoshi NN Lee AH Vallabhajosyula P Otipoby KL Rajewsky K Glimcher LH. Plasma cell differentiation and the unfolded protein response intersect at the transcription factor XBP-1. Nat Immunol (2003) 4(4):321–9. 10.1038/ni907 12612580

[135] Iwakoshi NN Pypaert M Glimcher LH. The transcription factor XBP-1 is essential for the development and survival of dendritic cells. J Exp Med (2007) 204(10):2267–75. 10.1084/jem.20070525 17875675 PMC2118458

[136] Brunsing R Omori SA Weber F Bicknell A Friend L Rickert R B- and T-cell development both involve activity of the unfolded protein response pathway. J Biol Chem (2008) 283(26):17954–61. 10.1074/jbc.M801395200 18375386

[137] Kamimura D Bevan MJ. Endoplasmic reticulum stress regulator XBP-1 contributes to effector CD8+ T cell differentiation during acute infection. J Immunol (2008) 181(8):5433–41. 10.4049/jimmunol.181.8.5433 18832700 PMC2776092

[138] Kemp KL Lin Z Zhao F Gao B Song J Zhang K The serine-threonine kinase inositol-requiring enzyme 1alpha (IRE1alpha) promotes IL-4 production in T helper cells. J Biol Chem (2013) 288(46):33272–82. 10.1074/jbc.M113.493171 24100031 PMC3829173

[139] Osorio F Tavernier SJ Hoffmann E Saeys Y Martens L Vetters J The unfolded-protein-response sensor IRE-1alpha regulates the function of CD8alpha+ dendritic cells. Nat Immunol (2014) 15(3):248–57. 10.1038/ni.2808 24441789

[140] Iwawaki T Akai R Kohno K Miura M. A transgenic mouse model for monitoring endoplasmic reticulum stress. Nat Med (2004) 10(1):98–102. 10.1038/nm1004-1014 14702639

[141] Cubillos-Ruiz JR Silberman PC Rutkowski MR Chopra S Perales-Puchalt A Song M ER stress sensor XBP1 controls anti-tumor immunity by disrupting dendritic cell homeostasis. Cell (2015) 161(7):1527–38. 10.1016/j.cell.2015.05.025 26073941 PMC4580135

[142] Tavernier SJ Osorio F Vandersarren L Vetters J Vanlangenakker N Van Isterdael G Regulated IRE1-dependent mRNA decay sets the threshold for dendritic cell survival. Nat Cell Biol (2017) 19(6):698–710. 10.1038/ncb3518 28459443 PMC5563826

[143] Bettigole SE Lis R Adoro S Lee AH Spencer LA Weller PF The transcription factor XBP1 is selectively required for eosinophil differentiation. Nat Immunol (2015) 16(8):829–37. 10.1038/ni.3225 26147683 PMC4577297

[144] Martinon F Chen X Lee AH Glimcher LH. TLR activation of the transcription factor XBP1 regulates innate immune responses in macrophages. Nat Immunol (2010) 11(5):411–8. 10.1038/ni.1857 20351694 PMC3113706

[145] Tufanli O Telkoparan Akillilar P Acosta-Alvear D Kocaturk B Onat UI Hamid SM Targeting IRE1 with small molecules counteracts progression of atherosclerosis. Proc Natl Acad Sci U S A (2017) 114(8):E1395–404. 10.1073/pnas.1621188114 28137856 PMC5338400

[146] Kurata M Yamazaki Y Kanno Y Ishibashi S Takahara T Kitagawa M Anti-apoptotic function of Xbp1 as an IL-3 signaling molecule in hematopoietic cells. Cell Death Dis (2011) 2:e118. 10.1038/cddis.2011.1 21368889 PMC3101701

[147] Dickhout JG Lhotak S Hilditch BA Basseri S Colgan SM Lynn EG Induction of the unfolded protein response after monocyte to macrophage differentiation augments cell survival in early atherosclerotic lesions. FASEB J (2011) 25(2):576–89. 10.1096/fj.10-159319 20966213

[148] Tohmonda T Yoda M Iwawaki T Matsumoto M Nakamura M Mikoshiba K IRE1alpha/XBP1-mediated branch of the unfolded protein response regulates osteoclastogenesis. J Clin Invest (2015) 125(8):3269–79. 10.1172/JCI76765 26193638 PMC4563737

[149] Hu R Chen ZF Yan J Li QF Huang Y Xu H Endoplasmic reticulum stress of neutrophils is required for ischemia/reperfusion-induced acute lung injury. J Immunol (2015) 195(10):4802–9. 10.4049/jimmunol.1500073 26475925 PMC4635566

[150] Mujaj S Gandhi M Vari F Nourse J Modulation of the unfolded protein response via XBP1 splicing: a novel mechanism that regulates natural killer cell effector function.(172.10). J Immunol (2012) 188(Suppl 1):172.10.

[151] Nam ST Park YH Kim HW Kim HS Lee D Lee MB Suppression of IgE-mediated mast cell activation and mouse anaphylaxis via inhibition of Syk activation by 8-formyl-7-hydroxy-4-methylcoumarin, 4mu8C. Toxicol Appl Pharmacol (2017) 332:25–31. 10.1016/j.taap.2017.07.015 28736076

[152] Eizirik DL Colli ML Ortis F. The role of inflammation in insulitis and beta-cell loss in type 1 diabetes. Nat Rev Endocrinol (2009) 5(4):219–26. 10.1038/nrendo.2009.21 19352320

[153] Gurzov EN Eizirik DL Bcl-2 proteins in diabetes: mitochondrial pathways of beta-cell death and dysfunction. Trends Cell Biol (2011) 21(7):424–31. 10.1016/j.tcb.2011.03.001 21481590

[154] Allagnat F Fukaya M Nogueira TC Delaroche D Welsh N Marselli L C/EBP homologous protein contributes to cytokine-induced pro-inflammatory responses and apoptosis in beta-cells. Cell Death Differ (2012) 19(11):1836–46. 10.1038/cdd.2012.67 22653339 PMC3469067

[155] Guo X Meng Y Sheng X Guan Y Zhang F Han Z Tunicamycin enhances human colon cancer cells to TRAIL-induced apoptosis by JNK-CHOP-mediated DR5 upregulation and the inhibition of the EGFR pathway. Anticancer Drugs (2017) 28(1):66–74. 10.1097/CAD.0000000000000431 27603596

[156] Minn AH Hafele C Shalev A. Thioredoxin-interacting protein is stimulated by glucose through a carbohydrate response element and induces beta-cell apoptosis. Endocrinology (2005) 146(5):2397–405. 10.1210/en.2004-1378 15705778

[157] Chen J Hui ST Couto FM Mungrue IN Davis DB Attie AD Thioredoxin-interacting protein deficiency induces Akt/Bcl-xL signaling and pancreatic beta-cell mass and protects against diabetes. FASEB J (2008) 22(10):3581–94. 10.1096/fj.08-111690 18552236 PMC2537437

[158] Lombardi A Tomer Y Interferon alpha impairs insulin production in human beta cells via endoplasmic reticulum stress. J Autoimmun (2017) 80:48–55. 10.1016/j.jaut.2017.02.002 28238527 PMC5758382

[159] Savic S Ouboussad L Dickie LJ Geiler J Wong C Doody GM TLR dependent XBP-1 activation induces an autocrine loop in rheumatoid arthritis synoviocytes. J Autoimmun (2014) 50:59–66. 10.1016/j.jaut.2013.11.002 24387801 PMC4012140

[160] Ospelt C Brentano F Rengel Y Stanczyk J Kolling C Tak PP Overexpression of toll-like receptors 3 and 4 in synovial tissue from patients with early rheumatoid arthritis: toll-like receptor expression in early and longstanding arthritis. Arthritis Rheum (2008) 58(12):3684–92. 10.1002/art.24140 19035519

[161] Kabala PA Angiolilli C Yeremenko N Grabiec AM Giovannone B Pots D Endoplasmic reticulum stress cooperates with toll-like receptor ligation in driving activation of rheumatoid arthritis fibroblast-like synoviocytes. Arthritis Res Ther (2017) 19(1):207. 10.1186/s13075-017-1386-x 28923079 PMC5604427

[162] Wang J Cheng Q Wang X Zu B Xu J Xu Y Deficiency of IRE1 and PERK signal pathways in systemic lupus erythematosus. Am J Med Sci (2014) 348(6):465–73. 10.1097/MAJ.0000000000000328 25226532

[163] Guo G Meng Y Tan W Xia Y Cheng C Chen X Induction of apoptosis coupled to endoplasmic reticulum stress through regulation of CHOP and JNK in bone marrow mesenchymal stem cells from patients with systemic lupus erythematosus. J Immunol Res (2015) 2015:183738 10.1155/2015/183738 26090483 PMC4452351

[164] Toosi S Orlow SJ Manga P. Vitiligo-inducing phenols activate the unfolded protein response in melanocytes resulting in upregulation of IL6 and IL8. J Invest Dermatol (2012) 132(11):2601–9. 10.1038/jid.2012.181 22696056 PMC3443495

[165] Alghamdi KM Khurrum H Taieb A Ezzedine K Treatment of generalized vitiligo with anti-TNF-alpha agents. J Drugs Dermatol (2012) 11(4):534–9. 22453596

[166] Li S Zhu G Yang Y Jian Z Guo S Dai W Oxidative stress drives CD8(+) T-cell skin trafficking in patients with vitiligo through CXCL16 upregulation by activating the unfolded protein response in keratinocytes. J Allergy Clin Immunol (2017) 140(1):177–89.e9. 10.1016/j.jaci.2016.10.013 27826097

[167] He Y Sun S Sha H Liu Z Yang L Xue Z Emerging roles for XBP1, a sUPeR transcription factor. Gene Expr (2010) 15(1):13–25. 10.3727/105221610X12819686555051 21061914 PMC3374844

[168] Kaser A Zeissig S Blumberg RS. Inflammatory bowel disease. Annu Rev Immunol (2010) 28:573–621. 10.1146/annurev-immunol-030409-101225 20192811 PMC4620040

[169] Adolph TE Tomczak MF Niederreiter L Ko HJ Bock J Martinez-Naves E Paneth cells as a site of origin for intestinal inflammation. Nature (2013) 503(7475):272–6. 10.1038/nature12599 24089213 PMC3862182

[170] Lenna S Farina AG Martyanov V Christmann RB Wood TA Farber HW Increased expression of endoplasmic reticulum stress and unfolded protein response genes in peripheral blood mononuclear cells from patients with limited cutaneous systemic sclerosis and pulmonary arterial hypertension. Arthritis Rheum (2013) 65(5):1357–66. 10.1002/art.37891 23400395 PMC3636187

[171] Heindryckx F Binet F Ponticos M Rombouts K Lau J Kreuger J Endoplasmic reticulum stress enhances fibrosis through IRE1alpha-mediated degradation of miR-150 and XBP-1 splicing. EMBO Mol Med (2016) 8(7):729–44. 10.15252/emmm.201505925 27226027 PMC4931288

[172] Li Y Schwabe RF DeVries-Seimon T Yao PM Gerbod-Giannone MC Tall AR Free cholesterol-loaded macrophages are an abundant source of tumor necrosis factor-alpha and interleukin-6: model of NF-kappaB- and map kinase-dependent inflammation in advanced atherosclerosis. J Biol Chem (2005) 280(23):21763–72. 10.1074/jbc.M501759200 15826936

[173] Navid F Colbert RA. Causes and consequences of endoplasmic reticulum stress in rheumatic disease. Nat Rev Rheumatol (2017) 13(1):25–40. 10.1038/nrrheum.2016.192 27904144

[174] Eferl R Wagner EF AP-1: a double-edged sword in tumorigenesis. Nat Rev Cancer (2003) 3(11):859–68. 10.1038/nrc1209 14668816

[175] Keestra-Gounder AM Byndloss MX Seyffert N Young BM Chavez-Arroyo A Tsai AY NOD1 and NOD2 signalling links ER stress with inflammation. Nature (2016) 532(7599):394–7. 10.1038/nature17631 27007849 PMC4869892

[176] Hu P Han Z Couvillon AD Kaufman RJ Exton JH. Autocrine tumor necrosis factor alpha links endoplasmic reticulum stress to the membrane death receptor pathway through IRE1alpha-mediated NF-kappaB activation and down-regulation of TRAF2 expression. Mol Cell Biol (2006) 26(8):3071–84. 10.1128/MCB.26.8.3071-3084.2006 16581782 PMC1446932

[177] Tam AB Mercado EL Hoffmann A Niwa M ER stress activates NF-kappaB by integrating functions of basal IKK activity, IRE1 and PERK. PLoS One (2012) 7(10):e45078 10.1371/journal.pone.0045078 23110043 PMC3482226

[178] Smith JA Turner MJ DeLay ML Klenk EI Sowders DP Colbert RA. Endoplasmic reticulum stress and the unfolded protein response are linked to synergistic IFN-beta induction via X-box binding protein 1. Eur J Immunol (2008) 38(5):1194–203. 10.1002/eji.200737882 18412159 PMC2838478

[179] Beisel C Ziegler S Martrus Zapater G Chapel A Griesbeck M Hildebrandt H TLR7-mediated activation of XBP1 correlates with the IFNalpha production in humans. Cytokine (2017) 94:55–8. 10.1016/j.cyto.2017.04.006 28408069

[180] Reed M Morris SH Owczarczyk AB Lukacs NW. Deficiency of autophagy protein Map1-LC3b mediates IL-17-dependent lung pathology during respiratory viral infection via ER stress-associated IL-1. Mucosal Immunol (2015) 8(5):1118–30. 10.1038/mi.2015.3 25669150 PMC4532659

[181] Zhou X Paulsson G Stemme S Hansson GK. Hypercholesterolemia is associated with a T helper (Th) 1/Th2 switch of the autoimmune response in atherosclerotic apo E-knockout mice. J Clin Invest (1998) 101(8):1717–25. 10.1172/JCI1216 9541503 PMC508754

[182] Ait-Oufella H Taleb S Mallat Z Tedgui A. Recent advances on the role of cytokines in atherosclerosis. Arterioscler Thromb Vasc Biol (2011) 31(5):969–79. 10.1161/ATVBAHA.110.207415 21508343

[183] Guo H Callaway JB Ting JP Inflammasomes: mechanism of action, role in disease, and therapeutics. Nat Med (2015) 21(7):677–87. 10.1038/nm.3893 26121197 PMC4519035

[184] Chen L Li Q She T Li H Yue Y Gao S IRE1alpha-XBP1 signaling pathway, a potential therapeutic target in multiple myeloma. Leuk Res (2016) 49:7–12. 10.1016/j.leukres.2016.07.006 27518808

[185] Cho JA Lee AH Platzer B Cross BC Gardner BM De Luca H The unfolded protein response element IRE1alpha senses bacterial proteins invading the ER to activate RIG-I and innate immune signaling. Cell Host Microbe (2013) 13(5):558–69. 10.1016/j.chom.2013.03.011 23684307 PMC3766372

[186] Anthony TG Wek RC. TXNIP switches tracks toward a terminal UPR. Cell Metab (2012) 16(2):135–7. 10.1016/j.cmet.2012.07.012 22883225

[187] Broderick L De Nardo D Franklin BS Hoffman HM Latz E. The inflammasomes and autoinflammatory syndromes. Annu Rev Pathol (2015) 10:395–424. 10.1146/annurev-pathol-012414-040431 25423351

[188] Zhang K Shen X Wu J Sakaki K Saunders T Rutkowski DT Endoplasmic reticulum stress activates cleavage of CREBH to induce a systemic inflammatory response. Cell (2006) 124(3):587–99. 10.1016/j.cell.2005.11.040 16469704

[189] Heemels MT Ploegh H. Generation, translocation, and presentation of MHC class I-restricted peptides. Annu Rev Biochem (1995) 64:463–91. 10.1146/annurev.bi.64.070195.002335 7574490

[190] Lankat-Buttgereit B Tampe R. The transporter associated with antigen processing: function and implications in human diseases. Physiol Rev (2002) 82(1):187–204. 10.1152/physrev.00025.2001 11773612

[191] Yadav D Ngolab J Lim RS Krishnamurthy S Bui JD. Cutting edge: down-regulation of MHC class I-related chain A on tumor cells by IFN-gamma-induced microRNA. J Immunol (2009) 182(1):39–43. 10.4049/jimmunol.182.1.39 19109132 PMC2714222

[192] Bartoszewski R Brewer JW Rab A Crossman DK Bartoszewska S Kapoor N The unfolded protein response (UPR)-activated transcription factor X-box-binding protein 1 (XBP1) induces microRNA-346 expression that targets the human antigen peptide transporter 1 (TAP1) mRNA and governs immune regulatory genes. J Biol Chem (2011) 286(48):41862–70. 10.1074/jbc.M111.304956 22002058 PMC3308892

[193] Todd DJ Lee AH Glimcher LH. The endoplasmic reticulum stress response in immunity and autoimmunity. Nat Rev Immunol (2008) 8(9):663–74. 10.1038/nri2359 18670423

[194] Wang J Takeuchi T Tanaka S Kubo SK Kayo T Lu D A mutation in the insulin 2 gene induces diabetes with severe pancreatic beta-cell dysfunction in the Mody mouse. J Clin Invest (1999) 103(1):27–37. 10.1172/JCI4431 9884331 PMC407861

[195] Ron D Proteotoxicity in the endoplasmic reticulum: lessons from the Akita diabetic mouse. J Clin Invest (2002) 109(4):443–5. 10.1172/JCI0215020 11854314 PMC150880

[196] Ghosh R Wang L Wang ES Perera BG Igbaria A Morita S Allosteric inhibition of the IRE1alpha RNase preserves cell viability and function during endoplasmic reticulum stress. Cell (2014) 158(3):534–48. 10.1016/j.cell.2014.07.002 25018104 PMC4244221

[197] Chatzikyriakidou A Voulgari PV Drosos AA. What is the role of HLA-B27 in spondyloarthropathies? Autoimmun Rev (2011) 10(8):464–8. 10.1016/j.autrev.2011.01.011 21296192

[198] Chen B Li J He C Li D Tong W Zou Y Role of HLA-B27 in the pathogenesis of ankylosing spondylitis (review). Mol Med Rep (2017) 15(4):1943–51. 10.3892/mmr.2017.6248 28259985 PMC5364987

[199] Marker-Hermann E Meyer zum Buschenfelde KH Wildner G. HLA-B27-derived peptides as autoantigens for T lymphocytes in ankylosing spondylitis. Arthritis Rheum (1997) 40(11):2047–54. 10.1002/art.1780401118 9365095

[200] Colbert RA Tran TM Layh-Schmitt G. HLA-B27 misfolding and ankylosing spondylitis. Mol Immunol (2014) 57(1):44–51. 10.1016/j.molimm.2013.07.013 23993278 PMC3857088

[201] DeLay ML Turner MJ Klenk EI Smith JA Sowders DP Colbert RA. HLA-B27 misfolding and the unfolded protein response augment interleukin-23 production and are associated with Th17 activation in transgenic rats. Arthritis Rheum (2009) 60(9):2633–43. 10.1002/art.24763 19714651 PMC2893331

[202] Wang Q Franks HA Lax SJ El Refaee M Malecka A Shah S The ataxia telangiectasia mutated kinase pathway regulates IL-23 expression by human dendritic cells. J Immunol (2013) 190(7):3246–55. 10.4049/jimmunol.1201484 23460736 PMC3672964

[203] Marquez S Fernandez JJ Teran-Cabanillas E Herrero C Alonso S Azogil A Endoplasmic reticulum stress sensor IRE1alpha enhances IL-23 expression by human dendritic cells. Front Immunol (2017) 8:639 10.3389/fimmu.2017.00639 28674530 PMC5475432

[204] Layh-Schmitt G Yang EY Kwon G Colbert RA HLA-B27 alters the response to tumor necrosis factor alpha and promotes osteoclastogenesis in bone marrow monocytes from HLA-B27-transgenic rats. Arthritis Rheum (2013) 65(8):2123–31. 10.1002/art.38001 23666508 PMC3857096

[205] Herrera-Esparza R Herrera-van-Oostdam D Lopez-Robles E Avalos-Diaz E. The role of apoptosis in autoantibody production. Reumatismo (2007) 59(2):87–99. 17603689 10.4081/reumatismo.2007.87

[206] Wu J Li X Song W Fang Y Yu L Liu S The roles and applications of autoantibodies in progression, diagnosis, treatment and prognosis of human malignant tumours. Autoimmun Rev (2017) 16(12):1270–81. 10.1016/j.autrev.2017.10.012 29042252

[207] Hirota M Kitagaki M Itagaki H Aiba S. Quantitative measurement of spliced XBP1 mRNA as an indicator of endoplasmic reticulum stress. J Toxicol Sci (2006) 31(2):149–56. 10.2131/jts.31.149 16772704

[208] Blass S Union A Raymackers J Schumann F Ungethum U Muller-Steinbach S The stress protein BiP is overexpressed and is a major B and T cell target in rheumatoid arthritis. Arthritis Rheum (2001) 44(4):761–71. 10.1002/1529-0131(200104)44:4<761::AID-ANR132>3.0.CO;2-S 11315915

[209] Yoo SA You S Yoon HJ Kim DH Kim HS Lee K A novel pathogenic role of the ER chaperone GRP78/BiP in rheumatoid arthritis. J Exp Med (2012) 209(4):871–86. 10.1084/jem.20111783 22430489 PMC3328363

[210] Corrigall VM Bodman-Smith MD Fife MS Canas B Myers LK Wooley P The human endoplasmic reticulum molecular chaperone BiP is an autoantigen for rheumatoid arthritis and prevents the induction of experimental arthritis. J Immunol (2001) 166(3):1492–8. 10.4049/jimmunol.166.3.1492 11160188

[211] Roda G Sartini A Zambon E Calafiore A Marocchi M Caponi A Intestinal epithelial cells in inflammatory bowel diseases. World J Gastroenterol (2010) 16(34):4264–71. 10.3748/wjg.v16.i34.4264 20818809 PMC2937106

[212] Yamasaki S Yagishita N Tsuchimochi K Nishioka K Nakajima T. Rheumatoid arthritis as a hyper-endoplasmic-reticulum-associated degradation disease. Arthritis Res Ther (2005) 7(5):181–6. 10.1186/ar1808 16207344 PMC1257448

[213] Yagishita N Aratani S Leach C Amano T Yamano Y Nakatani K RING-finger type E3 ubiquitin ligase inhibitors as novel candidates for the treatment of rheumatoid arthritis. Int J Mol Med (2012) 30(6):1281–6. 10.3892/ijmm.2012.1129 22992760 PMC4042867

[214] Barrera MJ Aguilera S Castro I Cortes J Bahamondes V Quest AFG Pro-inflammatory cytokines enhance ERAD and ATF6alpha pathway activity in salivary glands of Sjogren’s syndrome patients. J Autoimmun (2016) 75:68–81. 10.1016/j.jaut.2016.07.006 27461470

[215] Kaneko M Yasui S Niinuma Y Arai K Omura T Okuma Y A different pathway in the endoplasmic reticulum stress-induced expression of human HRD1 and SEL1 genes. FEBS Lett (2007) 581(28):5355–60. 10.1016/j.febslet.2007.10.033 17967421

[216] Riedhammer C Weissert R. Antigen presentation, autoantigens, and immune regulation in multiple sclerosis and other autoimmune diseases. Front Immunol (2015) 6:322. 10.3389/fimmu.2015.00322 26136751 PMC4470263

[217] Saric T Chang SC Hattori A York IA Markant S Rock KL An IFN-gamma-induced aminopeptidase in the ER, ERAP1, trims precursors to MHC class I-presented peptides. Nat Immunol (2002) 3(12):1169–76. 10.1038/ni859 12436109

[218] Blum JS Wearsch PA Cresswell P. Pathways of antigen processing. Annu Rev Immunol (2013) 31:443–73. 10.1146/annurev-immunol-032712-095910 23298205 PMC4026165

[219] Sadasivan B Lehner PJ Ortmann B Spies T Cresswell P. Roles for calreticulin and a novel glycoprotein, tapasin, in the interaction of MHC class I molecules with TAP. Immunity (1996) 5(2):103–14. 10.1016/S1074-7613(00)80487-2 8769474

[220] Alsaleh G Suffert G Semaan N Juncker T Frenzel L Gottenberg JE Bruton’s tyrosine kinase is involved in miR-346-related regulation of IL-18 release by lipopolysaccharide-activated rheumatoid fibroblast-like synoviocytes. J Immunol (2009) 182(8):5088–97. 10.4049/jimmunol.0801613 19342689

[221] Sebastiani G Grieco FA Spagnuolo I Galleri L Cataldo D Dotta F. Increased expression of microRNA miR-326 in type 1 diabetic patients with ongoing islet autoimmunity. Diabetes Metab Res Rev (2011) 27(8):862–6. 10.1002/dmrr.1262 22069274

[222] Fu Y Nathan DM Li F Li X Faustman DL Defective major histocompatibility complex class I expression on lymphoid cells in autoimmunity. J Clin Invest (1993) 91(5):2301–7. 10.1172/JCI116459 8486790 PMC288235

[223] Nakamura MC Nakamura RM. Contemporary concepts of autoimmunity and autoimmune diseases. J Clin Lab Anal (1992) 6(5):275–89. 10.1002/jcla.1860060506 1403347

[224] Bielekova B Sung MH Kadom N Simon R McFarland H Martin R. Expansion and functional relevance of high-avidity myelin-specific CD4+ T cells in multiple sclerosis. J Immunol (2004) 172(6):3893–904. 10.4049/jimmunol.172.6.3893 15004197

[225] Arif S Tree TI Astill TP Tremble JM Bishop AJ Dayan CM Autoreactive T cell responses show proinflammatory polarization in diabetes but a regulatory phenotype in health. J Clin Invest (2004) 113(3):451–63. 10.1172/JCI19585 14755342 PMC324541

[226] Anaya JM Common mechanisms of autoimmune diseases (the autoimmune tautology). Autoimmun Rev (2012) 11(11):781–4. 10.1016/j.autrev.2012.02.002 22353402

[227] Gabrielli A Svegliati S Moroncini G Avvedimento EV. Pathogenic autoantibodies in systemic sclerosis. Curr Opin Immunol (2007) 19(6):640–5. 10.1016/j.coi.2007.11.004 18083509

[228] Jaberi-Douraki M Schnell S Pietropaolo M Khadra A. Unraveling the contribution of pancreatic beta-cell suicide in autoimmune type 1 diabetes. J Theor Biol (2015) 375:77–87. 10.1016/j.jtbi.2014.05.003 24831415 PMC4232492

[229] Kahaly GJ Hansen MP. Type 1 diabetes associated autoimmunity. Autoimmun Rev (2016) 15(7):644–8. 10.1016/j.autrev.2016.02.017 26903475

[230] Seino Y Nanjo K Tajima N Kadowaki T Kashiwagi A Araki E Report of the committee on the classification and diagnostic criteria of diabetes mellitus. J Diabetes Investig (2010) 1(5):212–28. 10.1111/j.2040-1124.2010.00074.x 24843435 PMC4020724

[231] Sun J Cui J He Q Chen Z Arvan P Liu M. Proinsulin misfolding and endoplasmic reticulum stress during the development and progression of diabetes. Mol Aspects Med (2015) 42:105–18. 10.1016/j.mam.2015.01.001 25579745 PMC4404191

[232] Marroqui L Dos Santos RS Op de Beeck A Coomans de Brachene A Marselli L Marchetti P Interferon-alpha mediates human beta cell HLA class I overexpression, endoplasmic reticulum stress and apoptosis, three hallmarks of early human type 1 diabetes. Diabetologia (2017) 60(4):656–67. 10.1007/s00125-016-4201-3 28062922

[233] Lee SH Wray NR Goddard ME Visscher PM Estimating missing heritability for disease from genome-wide association studies. Am J Hum Genet (2011) 88(3):294–305. 10.1016/j.ajhg.2011.02.002 21376301 PMC3059431

[234] Geenen V. Thymus and type 1 diabetes: an update. Diabetes Res Clin Pract (2012) 98(1):26–32. 10.1016/j.diabres.2012.05.023 22717497

[235] Hasnain SZ Prins JB McGuckin MA Oxidative and endoplasmic reticulum stress in beta-cell dysfunction in diabetes. J Mol Endocrinol (2016) 56(2):R33–54. 10.1530/JME-15-0232 26576641

[236] Cnop M Welsh N Jonas JC Jorns A Lenzen S Eizirik DL. Mechanisms of pancreatic beta-cell death in type 1 and type 2 diabetes: many differences, few similarities. Diabetes (2005) 54(Suppl 2):S97–107. 10.2337/diabetes.54.suppl_2.S97 16306347

[237] Hara T Mahadevan J Kanekura K Hara M Lu S Urano F Calcium efflux from the endoplasmic reticulum leads to beta-cell death. Endocrinology (2014) 155(3):758–68. 10.1210/en.2013-1519 24424032 PMC3929724

[238] Brozzi F Eizirik DL. ER stress and the decline and fall of pancreatic beta cells in type 1 diabetes. Ups J Med Sci (2016) 121(2):133–9. 10.3109/03009734.2015.1135217 26899404 PMC4900073

[239] Weir GC Bonner-Weir S. Glucose driven changes in beta cell identity are important for function and possibly autoimmune vulnerability during the progression of type 1 diabetes. Front Genet (2017) 8:2. 10.3389/fgene.2017.00002 28174593 PMC5258704

[240] Bernales S Papa FR Walter P. Intracellular signaling by the unfolded protein response. Annu Rev Cell Dev Biol (2006) 22:487–508. 10.1146/annurev.cellbio.21.122303.120200 16822172

[241] Marhfour I Lopez XM Lefkaditis D Salmon I Allagnat F Richardson SJ Expression of endoplasmic reticulum stress markers in the islets of patients with type 1 diabetes. Diabetologia (2012) 55(9):2417–20. 10.1007/s00125-012-2604-3 22699564

[242] Delong T Wiles TA Baker RL Bradley B Barbour G Reisdorph R Pathogenic CD4 T cells in type 1 diabetes recognize epitopes formed by peptide fusion. Science (2016) 351(6274):711–4. 10.1126/science.aad2791 26912858 PMC4884646

[243] Lipson KL Fonseca SG Ishigaki S Nguyen LX Foss E Bortell R Regulation of insulin biosynthesis in pancreatic beta cells by an endoplasmic reticulum-resident protein kinase IRE1. Cell Metab (2006) 4(3):245–54. 10.1016/j.cmet.2006.07.007 16950141

[244] Hassler JR Scheuner DL Wang S Han J Kodali VK Li P The IRE1alpha/XBP1s pathway is essential for the glucose response and protection of beta cells. PLoS Biol (2015) 13(10):e1002277 10.1371/journal.pbio.1002277 26469762 PMC4607427

[245] Lee AH Heidtman K Hotamisligil GS Glimcher LH Dual and opposing roles of the unfolded protein response regulated by IRE1alpha and XBP1 in proinsulin processing and insulin secretion. Proc Natl Acad Sci U S A (2011) 108(21):8885–90. 10.1073/pnas.1105564108 21555585 PMC3102350

[246] Jonas JC Bensellam M Duprez J Elouil H Guiot Y Pascal SM. Glucose regulation of islet stress responses and beta-cell failure in type 2 diabetes. Diabetes Obes Metab (2009) 11(Suppl 4):65–81. 10.1111/j.1463-1326.2009.01112.x 19817790

[247] Liang Y Xu WD Peng H Pan HF Ye DQ. SOCS signaling in autoimmune diseases: molecular mechanisms and therapeutic implications. Eur J Immunol (2014) 44(5):1265–75. 10.1002/eji.201344369 24595859

[248] Allagnat F Christulia F Ortis F Pirot P Lortz S Lenzen S Sustained production of spliced X-box binding protein 1 (XBP1) induces pancreatic beta cell dysfunction and apoptosis. Diabetologia (2010) 53(6):1120–30. 10.1007/s00125-010-1699-7 20349222

[249] Meyerovich K Ortis F Allagnat F Cardozo AK. Endoplasmic reticulum stress and the unfolded protein response in pancreatic islet inflammation. J Mol Endocrinol (2016) 57(1):R1–17. 10.1530/JME-15-0306 27067637

[250] Grieco FA Vendrame F Spagnuolo I Dotta F. Innate immunity and the pathogenesis of type 1 diabetes. Semin Immunopathol (2011) 33(1):57–66. 10.1007/s00281-010-0206-z 20383637

[251] Zhou Y Lee J Reno CM Sun C Park SW Chung J Regulation of glucose homeostasis through a XBP-1-FoxO1 interaction. Nat Med (2011) 17(3):356–65. 10.1038/nm.2293 21317886 PMC3897616

[252] Miani M Colli ML Ladriere L Cnop M Eizirik DL Mild endoplasmic reticulum stress augments the proinflammatory effect of IL-1beta in pancreatic rat beta-cells via the IRE1alpha/XBP1s pathway. Endocrinology (2012) 153(7):3017–28. 10.1210/en.2011-2090 22529213

[253] Yang C Diiorio P Jurczyk A O’Sullivan-Murphy B Urano F Bortell R. Pathological endoplasmic reticulum stress mediated by the IRE1 pathway contributes to pre-insulitic beta cell apoptosis in a virus-induced rat model of type 1 diabetes. Diabetologia (2013) 56(12):2638–46. 10.1007/s00125-013-3044-4 24121653 PMC4845659

[254] Wali JA Gurzov EN Fynch S Elkerbout L Kay TW Masters SL Activation of the NLRP3 inflammasome complex is not required for stress-induced death of pancreatic islets. PLoS One (2014) 9(11):e113128. 10.1371/journal.pone.0113128 25405767 PMC4236141

[255] Wang J Song MY Lee JY Kwon KS Park BH The NLRP3 inflammasome is dispensable for ER stress-induced pancreatic beta-cell damage in Akita mice. Biochem Biophys Res Commun (2015) 466(3):300–5. 10.1016/j.bbrc.2015.09.009 26361146

[256] Youm YH Adijiang A Vandanmagsar B Burk D Ravussin A Dixit VD. Elimination of the NLRP3-ASC inflammasome protects against chronic obesity-induced pancreatic damage. Endocrinology (2011) 152(11):4039–45. 10.1210/en.2011-1326 21862613 PMC3199005

[257] Cardozo AK Ortis F Storling J Feng YM Rasschaert J Tonnesen M Cytokines downregulate the sarcoendoplasmic reticulum pump Ca2+ ATPase 2b and deplete endoplasmic reticulum Ca2+, leading to induction of endoplasmic reticulum stress in pancreatic beta-cells. Diabetes (2005) 54(2):452–61. 10.2337/diabetes.54.2.452 15677503

[258] Bijlsma JW. Disease control with glucocorticoid therapy in rheumatoid arthritis. Rheumatology (Oxford) (2012) 51(Suppl 4):iv9–13. 10.1093/rheumatology/kes086 22685274

[259] Bartok B Firestein GS. Fibroblast-like synoviocytes: key effector cells in rheumatoid arthritis. Immunol Rev (2010) 233(1):233–55. 10.1111/j.0105-2896.2009.00859.x 20193003 PMC2913689

[260] Yamasaki S Yagishita N Tsuchimochi K Kato Y Sasaki T Amano T Resistance to endoplasmic reticulum stress is an acquired cellular characteristic of rheumatoid synovial cells. Int J Mol Med (2006) 18(1):113–7. 10.3892/ijmm.18.1.113 16786162

[261] Elshabrawy HA Essani AE Szekanecz Z Fox DA Shahrara S. TLRs, future potential therapeutic targets for RA. Autoimmun Rev (2017) 16(2):103–13. 10.1016/j.autrev.2016.12.003 27988432 PMC5290220

[262] Radstake TR Roelofs MF Jenniskens YM Oppers-Walgreen B van Riel PL Barrera P Expression of toll-like receptors 2 and 4 in rheumatoid synovial tissue and regulation by proinflammatory cytokines interleukin-12 and interleukin-18 via interferon-gamma. Arthritis Rheum (2004) 50(12):3856–65. 10.1002/art.20678 15593217

[263] Auf G Jabouille A Guerit S Pineau R Delugin M Bouchecareilh M Inositol-requiring enzyme 1alpha is a key regulator of angiogenesis and invasion in malignant glioma. Proc Natl Acad Sci U S A (2010) 107(35):15553–8. 10.1073/pnas.0914072107 20702765 PMC2932600

[264] Sokolove J Zhao X Chandra PE Robinson WH Immune complexes containing citrullinated fibrinogen costimulate macrophages via toll-like receptor 4 and Fcgamma receptor. Arthritis Rheum (2011) 63(1):53–62. 10.1002/art.30081 20954191 PMC3015008

[265] Shi B Huang Q Tak PP Vervoordeldonk MJ Huang CC Dorfleutner A SNAPIN: an endogenous toll-like receptor ligand in rheumatoid arthritis. Ann Rheum Dis (2012) 71(8):1411–7. 10.1136/annrheumdis-2011-200899 22523426

[266] Qiu Q Zheng Z Chang L Zhao YS Tan C Dandekar A Toll-like receptor-mediated IRE1alpha activation as a therapeutic target for inflammatory arthritis. EMBO J (2013) 32(18):2477–90. 10.1038/emboj.2013.183 23942232 PMC3770952

[267] Gao B Lee SM Chen A Zhang J Zhang DD Kannan K Synoviolin promotes IRE1 ubiquitination and degradation in synovial fibroblasts from mice with collagen-induced arthritis. EMBO Rep (2008) 9(5):480–5. 10.1038/embor.2008.37 18369366 PMC2373369

[268] Ko JS Koh JM So JS Jeon YK Kim HY Chung DH Palmitate inhibits arthritis by inducing t-bet and gata-3 mRNA degradation in iNKT cells via IRE1alpha-dependent decay. Sci Rep (2017) 7(1):14940 10.1038/s41598-017-14780-4 29097726 PMC5668299

[269] El-Gabalawy H. The preclinical stages of RA: lessons from human studies and animal models. Best Pract Res Clin Rheumatol (2009) 23(1):49–58. 10.1016/j.berh.2008.11.004 19233045

[270] Amano T Yamasaki S Yagishita N Tsuchimochi K Shin H Kawahara K Synoviolin/Hrd1, an E3 ubiquitin ligase, as a novel pathogenic factor for arthropathy. Genes Dev (2003) 17(19):2436–49. 10.1101/gad.1096603 12975321 PMC218080

[271] Yagishita N Yamasaki S Nishioka K Nakajima T. Synoviolin, protein folding and the maintenance of joint homeostasis. Nat Clin Pract Rheumatol (2008) 4(2):91–7. 10.1038/ncprheum0699 18235538

[272] Frieri M Stampfl H. Systemic lupus erythematosus and atherosclerosis: review of the literature. Autoimmun Rev (2016) 15(1):16–21. 10.1016/j.autrev.2015.08.007 26299985

[273] Weidenbusch M Kulkarni OP Anders HJ. The innate immune system in human systemic lupus erythematosus. Clin Sci (Lond) (2017) 131(8):625–34. 10.1042/CS20160415 28351959

[274] Hirabayashi Y Oka Y Ikeda T Fujii H Ishii T Sasaki T The endoplasmic reticulum stress-inducible protein, Herp, is a potential triggering antigen for anti-DNA response. J Immunol (2010) 184(6):3276–83. 10.4049/jimmunol.0900670 20147634

[275] Todd DJ McHeyzer-Williams LJ Kowal C Lee AH Volpe BT Diamond B XBP1 governs late events in plasma cell differentiation and is not required for antigen-specific memory B cell development. J Exp Med (2009) 206(10):2151–9. 10.1084/jem.20090738 19752183 PMC2757870

[276] Xavier RJ Podolsky DK. Unravelling the pathogenesis of inflammatory bowel disease. Nature (2007) 448(7152):427–34. 10.1038/nature06005 17653185

[277] Nee J Feuerstein JD. Optimizing the care and health of women with inflammatory bowel disease. Gastroenterol Res Pract (2015) 2015:435820. 10.1155/2015/435820 26089868 PMC4454754

[278] Wen Z Fiocchi C. Inflammatory bowel disease: autoimmune or immune-mediated pathogenesis? Clin Dev Immunol (2004) 11(3–4):195–204. 10.1080/17402520400004201 15559364 PMC2486322

[279] Wilson JC Furlano RI Jick SS Meier CR. Inflammatory bowel disease and the risk of autoimmune diseases. J Crohns Colitis (2016) 10(2):186–93. 10.1093/ecco-jcc/jjv193 26507860

[280] Halling ML Kjeldsen J Knudsen T Nielsen J Hansen LK. Patients with inflammatory bowel disease have increased risk of autoimmune and inflammatory diseases. World J Gastroenterol (2017) 23(33):6137–46. 10.3748/wjg.v23.i33.6137 28970729 PMC5597505

[281] Andersen NN Caspersen S Jess T Munkholm P Occurrence of demyelinating diseases after anti-TNFalpha treatment of inflammatory bowel disease: a Danish Crohn Colitis Database study. J Crohns Colitis (2008) 2(4):304–9. 10.1016/j.crohns.2008.04.001 21172228

[282] Li P Zheng Y Chen X Drugs for autoimmune inflammatory diseases: from small molecule compounds to anti-TNF biologics. Front Pharmacol (2017) 8:460 10.3389/fphar.2017.00460 28785220 PMC5506195

[283] Bogaert S De Vos M Olievier K Peeters H Elewaut D Lambrecht B Involvement of endoplasmic reticulum stress in inflammatory bowel disease: a different implication for colonic and ileal disease? PLoS One (2011) 6(10):e25589. 10.1371/journal.pone.0025589 22028783 PMC3196494

[284] Adolph TE Niederreiter L Blumberg RS Kaser A Endoplasmic reticulum stress and inflammation. Dig Dis (2012) 30(4):341–6. 10.1159/000338121 22796794 PMC3423328

[285] Tschurtschenthaler M Adolph TE Ashcroft JW Niederreiter L Bharti R Saveljeva S Defective ATG16L1-mediated removal of IRE1alpha drives Crohn’s disease-like ileitis. J Exp Med (2017) 214(2):401–22. 10.1084/jem.20160791 28082357 PMC5294857

[286] Heazlewood CK Cook MC Eri R Price GR Tauro SB Taupin D Aberrant mucin assembly in mice causes endoplasmic reticulum stress and spontaneous inflammation resembling ulcerative colitis. PLoS Med (2008) 5(3):e54. 10.1371/journal.pmed.0050054 18318598 PMC2270292

[287] Eri RD Adams RJ Tran TV Tong H Das I Roche DK An intestinal epithelial defect conferring ER stress results in inflammation involving both innate and adaptive immunity. Mucosal Immunol (2011) 4(3):354–64. 10.1038/mi.2010.74 21107311 PMC3130192

[288] Zhang HS Chen Y Fan L Xi QL Wu GH Li XX The endoplasmic reticulum stress sensor IRE1alpha in intestinal epithelial cells is essential for protecting against colitis. J Biol Chem (2015) 290(24):15327–36. 10.1074/jbc.M114.633560 25925952 PMC4463471

[289] Li M Zhang S Qiu Y He Y Chen B Mao R Upregulation of miR-665 promotes apoptosis and colitis in inflammatory bowel disease by repressing the endoplasmic reticulum stress components XBP1 and ORMDL3. Cell Death Dis (2017) 8(3):e2699 10.1038/cddis.2017.76 28333149 PMC5386569

[290] Zhang H Zhang Z Song G Tang X Song H Deng A Development of an XBP1 agonist, HLJ2, as a potential therapeutic agent for ulcerative colitis. Eur J Pharm Sci (2017) 109:56–64. 10.1016/j.ejps.2017.07.028 28757346

[291] Manga P Elbuluk N Orlow SJ Recent advances in understanding vitiligo. F1000Res (2016) 5 10.12688/f1000research.8976.1 PMC5017284 27635239

[292] Iannella G Greco A Didona D Didona B Granata G Manno A Vitiligo: pathogenesis, clinical variants and treatment approaches. Autoimmun Rev (2016) 15(4):335–43. 10.1016/j.autrev.2015.12.006 26724277

[293] Manga P Bis S Knoll K Perez B Orlow SJ. The unfolded protein response in melanocytes: activation in response to chemical stressors of the endoplasmic reticulum and tyrosinase misfolding. Pigment Cell Melanoma Res (2010) 23(5):627–34. 10.1111/j.1755-148X.2010.00718.x 20444203 PMC2939946

[294] Ren Y Yang S Xu S Gao M Huang W Gao T Genetic variation of promoter sequence modulates XBP1 expression and genetic risk for vitiligo. PLoS Genet (2009) 5(6):e1000523. 10.1371/journal.pgen.1000523 19543371 PMC2689933

[295] Oyarbide-Valencia K van den Boorn JG Denman CJ Li M Carlson JM Hernandez C Therapeutic implications of autoimmune vitiligo T cells. Autoimmun Rev (2006) 5(7):486–92. 10.1016/j.autrev.2006.03.012 16920575 PMC3462656

[296] van den Boorn JG Konijnenberg D Dellemijn TA van der Veen JP Bos JD Melief CJ Autoimmune destruction of skin melanocytes by perilesional T cells from vitiligo patients. J Invest Dermatol (2009) 129(9):2220–32. 10.1038/jid.2009.32 19242513

[297] Ferrari SM Fallahi P Santaguida G Virili C Ruffilli I Ragusa F Circulating CXCL10 is increased in non-segmental vitiligo, in presence or absence of autoimmune thyroiditis. Autoimmun Rev (2017) 16(9):946–50. 10.1016/j.autrev.2017.07.006 28698095

[298] Harris JE Harris TH Weninger W Wherry EJ Hunter CA Turka LA A mouse model of vitiligo with focused epidermal depigmentation requires IFN-gamma for autoreactive CD8(+) T-cell accumulation in the skin. J Invest Dermatol (2012) 132(7):1869–76. 10.1038/jid.2011.463 22297636 PMC3343174

[299] Wang XX Wang QQ Wu JQ Jiang M Chen L Zhang CF Increased expression of CXCR3 and its ligands in patients with vitiligo and CXCL10 as a potential clinical marker for vitiligo. Br J Dermatol (2016) 174(6):1318–26. 10.1111/bjd.14416 26801009

[300] Frisoli ML Harris JE. Vitiligo: mechanistic insights lead to novel treatments. J Allergy Clin Immunol (2017) 140(3):654–62. 10.1016/j.jaci.2017.07.011 28778794

[301] Gilbane AJ Denton CP Holmes AM. Scleroderma pathogenesis: a pivotal role for fibroblasts as effector cells. Arthritis Res Ther (2013) 15(3):215. 10.1186/ar4230 23796020 PMC4060542

[302] Pattanaik D Brown M Postlethwaite BC Postlethwaite AE. Pathogenesis of systemic sclerosis. Front Immunol (2015) 6:272. 10.3389/fimmu.2015.00272 26106387 PMC4459100

[303] Sriburi R Jackowski S Mori K Brewer JW XBP1: a link between the unfolded protein response, lipid biosynthesis, and biogenesis of the endoplasmic reticulum. J Cell Biol (2004) 167(1):35–41. 10.1083/jcb.200406136 15466483 PMC2172532

[304] Aghaei M Gharibdost F Zayeni H Akhlaghi M Sedighi S Rostamian AR Endothelin-1 in systemic sclerosis. Indian Dermatol Online J (2012) 3(1):14–6. 10.4103/2229-5178.93484 23130253 PMC3481917

[305] Jing J Dou TT Yang JQ Chen XB Cao HL Min M Role of endothelin-1 in the skin fibrosis of systemic sclerosis. Eur Cytokine Netw (2015) 26(1):10–4. 10.1684/ecn.2015.0360 25990837

[306] Gadea A Schinelli S Gallo V. Endothelin-1 regulates astrocyte proliferation and reactive gliosis via a JNK/c-Jun signaling pathway. J Neurosci (2008) 28(10):2394–408. 10.1523/JNEUROSCI.5652-07.2008 18322086 PMC2695974

[307] Wort SJ Ito M Chou PC Mc Master SK Badiger R Jazrawi E Synergistic induction of endothelin-1 by tumor necrosis factor alpha and interferon gamma is due to enhanced NF-kappaB binding and histone acetylation at specific kappaB sites. J Biol Chem (2009) 284(36):24297–305. 10.1074/jbc.M109.032524 19592490 PMC2782023

[308] Martinez-Miguel P Medrano-Andres D Griera-Merino M Ortiz A Rodriguez-Puyol M Rodriguez-Puyol D Tweak up-regulates endothelin-1 system in mouse and human endothelial cells. Cardiovasc Res (2017) 113(2):207–21. 10.1093/cvr/cvw239 28025386

[309] Bowman SJ Patient-reported outcomes including fatigue in primary Sjogren’s syndrome. Rheum Dis Clin North Am (2008) 34(4):949–962,ix. 10.1016/j.rdc.2008.08.010 18984414

[310] Burbelo PD Ambatipudi K Alevizos I Genome-wide association studies in Sjogren’s syndrome: what do the genes tell us about disease pathogenesis? Autoimmun Rev (2014) 13(7):756–61. 10.1016/j.autrev.2014.02.002 24657515 PMC4018829

[311] Masi G Annunziata P Sjogren’s syndrome and multiple sclerosis: two sides of the same coin? Autoimmun Rev (2016) 15(5):457–61. 10.1016/j.autrev.2016.01.013 26827908

[312] Bhattarai KR Lee HY Kim SH Kim HR Chae HJ. Ixeris dentata extract increases salivary secretion through the regulation of endoplasmic reticulum stress in a diabetes-induced xerostomia rat model. Int J Mol Sci (2018) 19(4):E1059. 10.3390/ijms19041059 29614832 PMC5979381

[313] Plate L Wiseman RL Regulating secretory proteostasis through the unfolded protein response: from function to therapy. Trends Cell Biol (2017) 27(10):722–37. 10.1016/j.tcb.2017.05.006 28647092 PMC5612838

[314] Meriggioli MN Sanders DB. Autoimmune myasthenia gravis: emerging clinical and biological heterogeneity. Lancet Neurol (2009) 8(5):475–90. 10.1016/S1474-4422(09)70063-8 19375665 PMC2730933

[315] Suzuki S Utsugisawa K Iwasa K Satoh T Nagane Y Yoshikawa H Autoimmunity to endoplasmic reticulum chaperone GRP94 in myasthenia gravis. J Neuroimmunol (2011) 237(1–2):87–92. 10.1016/j.jneuroim.2011.06.011 21774995

[316] Iwasa K Nambu Y Motozaki Y Furukawa Y Yoshikawa H Yamada M. Increased skeletal muscle expression of the endoplasmic reticulum chaperone GRP78 in patients with myasthenia gravis. J Neuroimmunol (2014) 273(1–2):72–6. 10.1016/j.jneuroim.2014.05.006 24882382

[317] Eletto D Dersh D Argon Y. GRP94 in ER quality control and stress responses. Semin Cell Dev Biol (2010) 21(5):479–85. 10.1016/j.semcdb.2010.03.004 20223290 PMC3676867

[318] Liu H Zeng Q Cui Y Yu L Zhao L Hou C The effects and underlying mechanism of excessive iodide on excessive fluoride-induced thyroid cytotoxicity. Environ Toxicol Pharmacol (2014) 38(1):332–40. 10.1016/j.etap.2014.06.008 25104093

[319] Wen G Ringseis R Eder K. Endoplasmic reticulum stress inhibits expression of genes involved in thyroid hormone synthesis and their key transcriptional regulators in FRTL-5 thyrocytes. PLoS One (2017) 12(11):e0187561. 10.1371/journal.pone.0187561 29095946 PMC5667865

[320] Feldman HC Tong M Wang L Meza-Acevedo R Gobillot TA Lebedev I Structural and functional analysis of the allosteric inhibition of IRE1alpha with ATP-competitive ligands. ACS Chem Biol (2016) 11(8):2195–205. 10.1021/acschembio.5b00940 27227314 PMC4992410

[321] Yang J Liu H Li L Shi W Yuan X Wu L. Structural insights into IRE1 functions in the unfolded protein response. Curr Med Chem (2016) 23(41):4706–16. 10.2174/0929867323666160927142349 27686654

[322] Wang L Perera BG Hari SB Bhhatarai B Backes BJ Seeliger MA Divergent allosteric control of the IRE1alpha endoribonuclease using kinase inhibitors. Nat Chem Biol (2012) 8(12):982–9. 10.1038/nchembio.1094 23086298 PMC3508346

[323] Chien W Ding LW Sun QY Torres-Fernandez LA Tan SZ Xiao J Selective inhibition of unfolded protein response induces apoptosis in pancreatic cancer cells. Oncotarget (2014) 5(13):4881–94. 10.18632/oncotarget.2051 24952679 PMC4148107

[324] Ming J Ruan S Wang M Ye D Fan N Meng Q A novel chemical, STF-083010, reverses tamoxifen-related drug resistance in breast cancer by inhibiting IRE1/XBP1. Oncotarget (2015) 6(38):40692–703. 10.18632/oncotarget.5827 26517687 PMC4747362

[325] Jiang D Niwa M Koong AC Targeting the IRE1alpha-XBP1 branch of the unfolded protein response in human diseases. Semin Cancer Biol (2015) 33:48–56. 10.1016/j.semcancer.2015.04.010 25986851 PMC4523453

[326] Zhao L Guo H Chen H Petersen RB Zheng L Peng A Effect of Liraglutide on endoplasmic reticulum stress in diabetes. Biochem Biophys Res Commun (2013) 441(1):133–8. 10.1016/j.bbrc.2013.10.026 24129189

[327] Wang N Mao L Yang L Zou J Liu K Liu M Resveratrol protects against early polymicrobial sepsis-induced acute kidney injury through inhibiting endoplasmic reticulum stress-activated NF-kappaB pathway. Oncotarget (2017) 8(22):36449–61. 10.18632/oncotarget.16860 28430592 PMC5482667

[328] Morita S Villalta SA Feldman HC Register AC Rosenthal W Hoffmann-Petersen IT Targeting ABL-IRE1alpha signaling spares ER-stressed pancreatic beta cells to reverse autoimmune diabetes. Cell Metab (2017) 25(4):883–97.e8. 10.1016/j.cmet.2017.03.018 28380378 PMC5497784

[329] Chae HJ Kim HR Xu C Bailly-Maitre B Krajewska M Krajewski S BI-1 regulates an apoptosis pathway linked to endoplasmic reticulum stress. Mol Cell (2004) 15(3):355–66. 10.1016/j.molcel.2004.06.038 15304216

[330] Lisbona F Rojas-Rivera D Thielen P Zamorano S Todd D Martinon F BAX inhibitor-1 is a negative regulator of the ER stress sensor IRE1alpha. Mol Cell (2009) 33(6):679–91. 10.1016/j.molcel.2009.02.017 19328063 PMC2818874

[331] Brozzi F Gerlo S Grieco FA Juusola M Balhuizen A Lievens S Ubiquitin D regulates IRE1alpha/c-Jun N-terminal kinase (JNK) protein-dependent apoptosis in pancreatic beta cells. J Biol Chem (2016) 291(23):12040–56. 10.1074/jbc.M115.704619 27044747 PMC4933257

[332] Pinkaew D Chattopadhyay A King MD Chunhacha P Liu Z Stevenson HL Fortilin binds IRE1alpha and prevents ER stress from signaling apoptotic cell death. Nat Commun (2017) 8(1):18 10.1038/s41467-017-00029-1 28550308 PMC5446404

